# Effect of Intrapersonal and Interpersonal Behavior Change Strategies on Physical Activity Among Older Adults: A Randomized Clinical Trial

**DOI:** 10.1001/jamanetworkopen.2024.0298

**Published:** 2024-02-29

**Authors:** Siobhan K. McMahon, Beth A. Lewis, Weihua Guan, Qi Wang, Shannon M. Hayes, Jean F. Wyman, Alexander J. Rothman

**Affiliations:** 1 School of Nursing, University of Minnesota, Minneapolis; 2 School of Kinesiology, University of Minnesota, Minneapolis; 3 School of Public Health, University of Minnesota, Minneapolis; 4 Department of Psychology, University of Minnesota, Minneapolis

## Abstract

**Importance:**

Despite guidelines that recommend physical activity (PA), little is known about which types of behavior change strategies (BCSs) effectively promote sustained increases in PA in older adults who are insufficiently active.

**Objective:**

To determine whether intrapersonal BCSs (eg, goal setting) or interpersonal BCSs (eg, peer-to-peer sharing or learning) combined with the Otago Exercise Program (17 strength and balance exercises and a walking program that are learned and individually tailored, with instruction to perform 3 times per week at home or location of choice) and a wearable PA monitor help older adults sustain increases in their PA.

**Design, Setting, and Participants:**

This 2 × 2 factorial randomized clinical trial (Community-Based Intervention Effects on Older Adults’ Physical Activity) of community-dwelling older adults 70 years or older with PA levels below minimum national PA guidelines was conducted in urban community centers. Dates of enrollment were from November 17, 2017, to June 15, 2021, with final follow-up assessments completed on September 2, 2022.

**Interventions:**

Participants were randomized to intrapersonal (eg, goal setting) BCSs, interpersonal (eg, problem-solving with peer-to-peer sharing and learning) BCSs, intrapersonal and interpersonal BCSs, or an attention control group. All interventions included a PA monitor and 8 weekly small-group meetings with discussion, practice, and instructions to implement the exercise program and relevant BCSs independently between meetings and after the intervention.

**Main Outcomes and Measures:**

The primary outcome was daily minutes of objectively measured total PA (light, moderate, or vigorous intensities) averaged over 7 to 10 days, measured at baseline and after the intervention at 1 week, 6 months, and 12 months.

**Results:**

Among 309 participants (mean [SD] age, 77.4 [5.0] years; 240 women [77.7%]), 305 (98.7%) completed the intervention, and 302 (97.7%) had complete data. Participants receiving PA interventions with interpersonal BCS components exhibited greater increases in total PA than did those who did not at 1 week (204 vs 177 PA minutes per day; adjusted difference, 27.1 [95% CI, 17.2-37.0]; *P* < .001), 6 months (195 vs 175 PA minutes per day; adjusted difference, 20.8 [95% CI, 10.0-31.6]; *P* < .001), and 12 months (195 vs 168 PA minutes per day; adjusted difference, 27.5 [95% CI, 16.2-38.8]; *P* < .001) after the intervention. Compared with participants who did not receive interventions with intrapersonal BCS components, participants who received intrapersonal BCSs exhibited no significant changes in total PA at 1 week (192 vs 190 PA minutes per day; adjusted difference, 1.8 [95% CI, −8.6 to 12.2]; *P* = .73), 6 months (183 vs 187 PA minutes per day; adjusted difference, −3.9 [95% CI, −15.0 to 7.1]; *P* = .49), or 12 months (177 vs 186 PA minutes per day; adjusted difference, −8.8 [95% CI, −20.5 to 2.9]; *P* = .14) after the intervention. Interactions between intrapersonal and interpersonal BCSs were not significant.

**Conclusions and Relevance:**

In this randomized clinical trial, older adults with low levels of PA who received interpersonal BCSs, the exercise program, and a PA monitor exhibited significant increases in their PA for up to 12 months after the intervention. Intrapersonal BCSs elicited no significant PA changes and did not interact with interpersonal BCSs. Our findings suggest that because effects of a PA intervention on sustained increases in older adults’ PA were augmented with interpersonal but not intrapersonal BCSs, approaches to disseminating and implementing the intervention should be considered.

**Trial Registration:**

ClinicalTrials.gov Identifier: NCT03326141

## Key Points

**Question:**

Can combining intrapersonal and/or interpersonal behavior change strategies (BCSs) with physical activity (PA) interventions promote sustained increases in total PA among community-dwelling older adults who are insufficiently active?

**Findings:**

In this randomized clinical trial of 309 community-based adults 70 years or older, those who received a PA intervention with interpersonal BCSs exhibited greater increases in their total PA for up to 12 months after the intervention than those who received a PA intervention without interpersonal BCSs. Conversely, participants who received PA interventions with intrapersonal BCSs exhibited no significant differences in PA than those who did not receive intrapersonal BCSs.

**Meaning:**

Interpersonal BCSs such as peer-to-peer experience sharing and learning should be considered in efforts and interventions that promote the sustained uptake of PA among older adults.

## Teaser

This randomized clinical trial examines whether community-dwelling older adults who were insufficiently active would increase their physical activity with intrapersonal and/or interpersonal behavior change strategies.

## Introduction

Low physical activity (PA) levels among older adults are associated with decreased physical function, disability, difficulty managing chronic conditions, and increased falls and related injuries.^1,2,3^ To counteract these problems, safe and effective^4^ aerobic, muscle-strengthening, and balance activities are recommended for all older adults,^3^ yet less than 16% meet minimum recommendations.^5^ One reason for the poor uptake of PA is limited knowledge regarding which types of behavior change strategies (BCSs) effectively promote sustained increases in PA in older adults.^3,6,7^ The current study presents results from a community-based randomized intervention factorial trial (Community-Based Intervention Effects on Older Adults’ Physical Activity), Ready Steady (RS) 3.0, that tested the relative effects of 2 types of BCSs, intrapersonal and interpersonal, on community-dwelling older adults’ PA.

Intrapersonal BCSs, such as problem-solving, goal setting, and action planning, are frequently included in PA interventions,^8,9^ designed to target putative psychosocial mechanisms through which an older person’s PA is theorized to increase (eg, self-efficacy, self-regulation).^10,11,12^ Interpersonal BCSs that involve peer-to-peer sharing and learning, such as social comparison and social support, are included in interventions less frequently^9,13,14,15^ and designed to target many of the same mechanisms^16,17^ as well as social processes (eg, support, networking, and engagement).^8,18,19,20^ Systematic reviews suggest both types of BCSs are associated with PA.^8,9,18,19,20,21,22^ However, experimental evidence regarding their main effects and interactions on total PA is lacking,^3,7,21^ except for a prior preliminary study (RS 2.0) that showed that interpersonal BCSs, but not intrapersonal BCSs, integrated into a PA intervention elicited increased PA after the intervention for up to 6 months.^23^

The present study, RS 3.0, used a randomized factorial design to address the gap in the literature and replicated the earlier RS 2.0 study^23^ but with a larger sample and a longer follow-up.^24^ It tested the main and interaction effects of intrapersonal and/or interpersonal BCSs integrated into an intervention comprising an evidence-based PA protocol and a wearable PA monitor (PAM) on older adults’ PA.

## Methods

### Study Design

The RS 3.0 trial was designed as a 2 × 2 full factorial randomized clinical trial. The factorial design and analyses enabled testing intrapersonal and interpersonal BCS components’ main and interaction effects when combined with the Otago Exercise Program^25^ and a wearable PAM. The exercise program consists of 17 strength and balance exercises and a walking program that are learned and individually tailored, with instruction to perform 3 times per week at home or location of choice.^26^ The approach was efficient because each effect estimate involved all 4 conditions.^27^ The trial design, protocol, and rationale are shown in Supplement 1 and a prior publication.^24^ Conducted in upper Midwest urban community centers in Minneapolis and Saint Paul, Minnesota, the study enrolled participants between November 17, 2017, and June 15, 2021, and all assessments were completed by September 2, 2022. Intervention delivery was paused between March 2020 and May 2021 due to the COVID-19 pandemic, but individual postintervention assessments continued using infection-prevention precautions. The University of Minnesota’s Institutional Review Board approved the study protocol, and participants provided written and verbal informed consent. This study followed the Consolidated Standards of Reporting Trials (CONSORT) reporting guideline for randomized controlled trials.

### Participants

Community-dwelling older adults were recruited using newspaper advertisements, online sources, printed flyers, presentations at community events, and word of mouth and were enrolled in 13 waves. Entrance criteria were being 70 years or older, not meeting the national guidelines recommended by the Physical Activity Guidelines for Americans Advisory Committee^3^ of at least 1 type of PA (eg, strength, balance, or aerobic), the ability to walk with or without an aid, 1 or more self-reported fall risks,^28^ having no lower-extremity injury or surgery within the last 6 weeks, and having no self-reported neurocognitive disorder or a score of less than 4 on the cognitive impairment screening tool with 6 items developed by Callahan and colleagues,^29^ in which scores range from 0 to 6, with higher scores (ie, 4 to 6) indicating a lower likelihood of cognitive impairment. The Exercise Assessment and Screening for You was also administered to ensure safety.^30^ Those who responded yes to questions about cardiovascular symptoms, frequent falls, or untreated dizziness obtained clearance from their primary care practitioner. We collected self-reported data on sex, race, and ethnicity to characterize populations for generalizability of findings. Self-reported race and ethnicity categories included Black or African American; Hispanic, Latino, or Spanish; White; and other race (Asian Indian, Chinese, Filipino, Indigenous, or some other race or ethnicity). Each participant received a wearable PAM (fitness tracker) and compensation of $70 for each assessment (up to $280 total).

### Study Procedure

#### Baseline Period

The baseline period included 3 contacts. During the first 2 contacts, participants completed baseline health and demographic questionnaires and received a new, wearable PAM.^31,32^ During the third baseline contact, participants completed self-reported questionnaires, and their accelerometer data from the previous 7 days were collected from wearable PAMs. They also received advanced, in-depth orientation and instructions for using the PAM.^24^

#### Randomization

Participants were randomized to interventions with the following components: intrapersonal BCS, the exercise program, and PAM; interpersonal BCS, the exercise program, and PAM; intrapersonal and interpersonal BCS, the exercise program, and PAM; or attention control information, the exercise program, and PAM. All interventions included 8 weekly small-group meetings with discussion, practice, and instructions to implement the exercise program and relevant BCSs independently between meetings and after the intervention.

**Figure 1.**
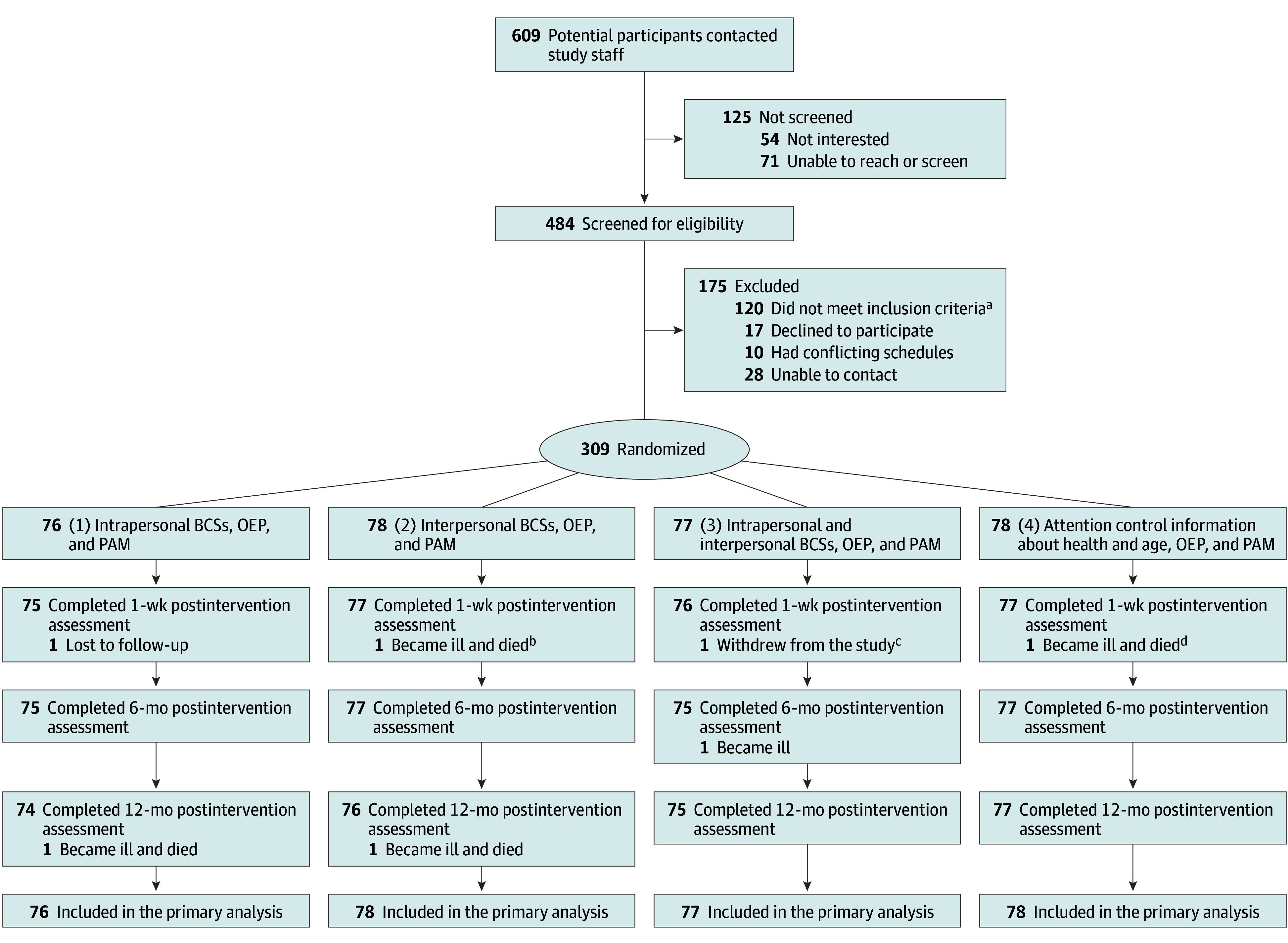
Diagram of Participant Flow Reasons for not meeting inclusion criteria (may have more than one) included currently meeting physical activity guidelines^3^ (n = 89), younger than 70 years (n = 21), unable to participate in the study for 12 or more months due to anticipated move (n = 8), unable to ambulate with or without a walking aid (n = 3), and recent lower extremity surgery or injury (n = 2). BCS indicates behavior change strategy; OEP, Otago Exercise Program; and PAM, physical activity monitor. ^a^For intervention condition 1, 1 participant was lost to follow-up because they were unable to be contacted after the intervention was started, and 1 participant became severely ill and died from causes unrelated to the study before the 12-month postintervention assessment. ^b^For intervention condition 2, 2 participants became ill and died from causes unrelated to the study: 1, after the intervention was started and 1, before the 12-month postintervention assessment. ^c^For intervention condition 3, 1 participant withdrew from the study before the 1-week postintervention assessment due to transportation difficulties, and 1 participant became severely ill from causes unrelated to the study before the 6-month postintervention assessment. ^d^For intervention condition 4, 1 participant became ill and died from causes unrelated to the study before the 1-week postintervention assessment.

Eligible participants who completed the baseline period were randomized to intervention conditions in a 1:1:1:1 ratio with the following components: (1) intrapersonal BCS, the exercise program, and PAM; (2) interpersonal BCS, the exercise program, and PAM; (3) intrapersonal and interpersonal BCS, the exercise program, and PAM; and (4) attention control information about health and age, the exercise program, and PAM (Figure 1). A total of 38 pairs of partners or friends eligible for the study and who requested to receive the intervention in the same small group were randomized together to minimize contamination between study conditions.^33^ To conceal random allocation sequences until interventions were assigned, the study analyst (Q.W.) generated 1 allocation sequence for each wave of 16 to 24 participants using SAS, version 9.4 (SAS Institute Inc) and provided access to the study manager after the study manager communicated that the wave was enrolled and completed baseline assessments. The study manager then assigned participants to interventions according to the random allocation sequence. Research staff responsible for assessments were masked to condition assignments and intervention content through the use of numeric codes for condition labels, the key to which they did not have access.

#### Intervention

Condition and meeting-specific curricula, manuals, and workbooks were created to guide interventionists and participants through the small-group intervention. The intrapersonal and interpersonal BCSs were considered experimental intervention components in conditions 1, 2, and 3. Information about popular health and age topics was provided as attention control content in condition 4, which contained no BCS. The evidence-based OEP^25,34^ and wearable PAMs^3,35^ were considered core components, integral to interventions in all conditions. In-depth details about each component’s delivery and behavior change content, links to behavior change techniques,^36^ their dosages, and what participants were encouraged to do with each BCS were previously reported.^37^

##### Experimental Components

In general, the interventionist delivered each BCS by first introducing it and then facilitating its practice during 2 intervention meetings. The meetings involved encouragement to build on, test, and implement it at home between intervention meetings and after intervention completion.^37^

The intrapersonal BCS experimental component consisted of 5 BCSs that incorporated personal reflection on PA-related experiences, beliefs, desires, and routines. These BCSs (highlighted in the Box) were selected based on theoretical and empirical evidence^19,38,39^ and targeted the putative psychosocial mechanisms of readiness, self-efficacy, and self-regulation.

Box. **Content of Experimental Intervention Components in Ready Steady 3.0^a^**

**Intrapersonal behavior change strategy intervention component**

1. Consider personal barriers (mind, body) to PA; identify and try possible solution(s) at home.
2. Develop personal PA goals that are specific, measurable, attainable, relevant, and time-bound.
3. Develop personally relevant PA action plans for accomplishing goals.
4. Self-assess progress toward PA goal attainment and action plan implementation; adjust either or both as needed.
5. Outline personal daily routine, imagine possibilities for adding a new PA habit to 1 existing, and try and experiment at home.

**Interpersonal behavior change strategy intervention component**

1. Discuss environmental barriers to PA (physical, social); identify and try possible solution(s) at home.
2. Compare experiences with peers about motivation for and performance of PA at home and effects of PA.
3. Peer discussion and brainstorm: environmental (physical, social) prompts and cues for PA; try at home.
4. Peer discussion about social support for PA, preferred types, and how they work; increase outside intervention.
5. Peer discussion about being role models for others (eg, family, friends, and neighbors); acknowledge.

Abbreviation: PA, physical activity.

^a^
Overall intervention dosage across all intervention conditions was 8 weeks with weekly 90-minute meetings, for a total of approximately 720 minutes of contact time. Each behavior change strategy was addressed for 10 to 15 minutes during 2 meetings, except for peer discussions about being role models for others, which was addressed at 1 meeting.

The interpersonal BCS experimental component consisted of 5 BCSs that incorporated peer-to-peer sharing and learning about PA-related motivations, experiences, and knowledge. These BCSs (highlighted in the Box) were selected based on theoretical and empirical evidence^14,15,38^ and targeted the putative psychosocial mechanisms of readiness, self-efficacy, self-regulation, and social support.

The condition that contained no intrapersonal or interpersonal BCS included educational attention control content. Participants received information about and discussed 1 health topic for 20 minutes at each meeting: safety during PA, falls, pain, nutritional supplements, sleep, hearing, memory, and vaccinations.^40^

##### Core Components

The exercise program, adapted for small groups,^41,42^ included the gradual introduction, demonstration, individualization, practice, and progression of 5 leg-strengthening and 12 balance-challenging exercises, plus encouragement to walk daily at one’s usual pace (Supplement 1). It also included instruction and encouragement to perform the PAs learned and practiced during intervention meetings at home or at a preferred location at least 2 or 3 times per week after the intervention.

Wearable PAMs were provided to each participant, with displays consistent with the self-monitoring feature of the exercise program and PA promotion guidelines.^29,30^ Support for learning about and using the device was provided throughout the study.^24,43^

### Outcomes and Measures

The primary outcome was the quantity of PA operationalized as daily minutes of total PA (light, moderate, and vigorous intensities) averaged over 7 to 10 days, measured objectively using accelerometers built into participants’ wearable PAMs.^32,44,45,46^ Total PA was measured by self-report using the PA Scale for the Elderly as a secondary source of data if adherence to wearing the PAM was low.^47,48^ Post hoc outcomes included additional objective indicators of PA averaged over 7 to 10 days: total PA operationalized as daily step count and moderate and vigorously intense PAs (MVPAs; aerobic movement fast and strenuous enough to burn off 3 to 6 times as much energy per minute than when sitting quietly and vigorous aerobic movement fast and strenuous enough to burn off ≥6 times as much energy per minute than when sitting quietly) operationalized as minutes of both combined.^49^

Outcomes were assessed at baseline and at 3 time points after the intervention: 1 week, 6 months, and 12 months. Research staff connected deidentified fitness tracker accounts for each participant to Fitabase (Small Steps Labs LLC), a wearable research data management platform that includes the validation of participant wear time and data through minute-level heart rate and intensity data.^50^ The staff instructed participants to wear the PAM on their nondominant wrist during waking hours for at least 7 days, synchronize it frequently, and charge it at least every 5 days. At baseline and in cases during postintervention assessments when participants did not have internet access or a fitness tracker–compatible phone, wearable PAMs were synchronized by research staff to a study touchscreen tablet personal computer.

Staff collected data from 7 to 10 days before assessment meetings to overlap with the administration of the PA Scale for the Elderly. They checked accelerometer data against the minimum validation criteria of 4 or more days, including a weekend; 10 or more hours per day of wear time; and nonwear time of 60 or more minutes of continuous 0 measurements of heart rate or intensity data. If minimum validation criteria were not met, participants were asked to continue wearing the PAM, and a follow-up assessment was scheduled. Except for participants who withdrew from the study (Figure 1), data from all participants met valid wear-time criteria across all time points with less than 1% at the minimum level of valid wear-time criteria.

### Sample Size

A target sample size of 308 was determined based on an expected 15% attrition, 80% power under a 2-tailed hypothesis test, and a significance level of *P* = .05 to detect main or interaction effects of intrapersonal and interpersonal BCS intervention components of at least 0.2 (Cohen *d*).^19,51^ Although small, this effect size is considered clinically meaningful in older people and translates to 10 to 13 additional minutes of PA per day, or 670 to 870 additional steps per day.^19^

### Statistical Analysis

All participants’ data were included in the study and analyzed according to their randomly assigned conditions. Analysis of covariance models were used to assess changes in each outcome at each postintervention time point, controlling for baseline values. The 2-level factors in models were receipt of the experimental components intrapersonal (conditions 1 and 3 vs conditions 2 and 4) or interpersonal (conditions 2 and 3 vs conditions 1 and 4). These factors were effect coded with 2 levels indicating exposure (yes, +1; no, −1).^27^ Separate multivariable models were run with interaction terms for intrapersonal and interpersonal factors for each postintervention assessment time point. The statistical significance of all tests was set at a 2-sided level of *P* = .05. All statistical calculations and analyses were performed using SAS, version 9.4 (SAS Institute Inc).

To assess potential clustering effects of partnered participants and intervention small-group membership, we extended analysis of covariance models with the outcome of mean (SE) daily total minutes of PA to include random variable terms for each. Cluster analysis results were congruent with analyses without the random-effects terms and presented in the eFigure and eTable 3 in Supplement 2.

## Results

### Enrollment and Participant Characteristics and Intervention Attendance

A total of 309 participants were enrolled in the study (Figure 1), of whom 305 (98.7%) completed the intervention, and 302 (97.7%) had complete data. Table 1^3,47,52,53^ presents participant characteristics at baseline. The mean (SD) age was 77.4 (5.0) years; 69 (22.3%) were men, and 240 (77.7%) were women. Among participants, 48 (15.5%) were Black or African American ; 255 (82.5%) were White , and 6 (1.9%) were categorized as other race; 7 (2.3%) identified as Hispanic, Latino, or Spanish ethnicity. The study included 185 college graduates (59.9%), 139 participants living alone (45.0%), 100 (32.4%) with cardiovascular disease, 104 (33.7%) with osteoporosis, 216 (69.9%) with arthritis, and 64 (20.7%) with diabetes. eTable 2 in Supplement 2 presents baseline characteristics of participants randomized with a partner. The mean (SD) number of intervention meetings completed by participants in study conditions 1, 2, 3, and 4 (Figure 1) were 7.2 (1.1), 7.1 (0.83), 7.3 (0.88), and 7.0 (0.96) of 8 group meetings, respectively. The overall mean (SD) valid accelerometer data during assessments were 9.5 (2.8) days and 18.3 (5.1) hours per day.

**Table 1.** Baseline Characteristics of Participants by Intervention Condition

Characteristic	Intervention conditions, No. (%)^a^			
	Intrapersonal behavior change strategies (n = 76)	Interpersonal behavior change strategies (n = 78)	Intrapersonal and interpersonal behavior change strategies (n = 77)	Attention control information about health and age (n = 78)
Age, mean (SD), y	77.3 (5.0)	77.7 (5.0)	76.6 (5.1)	77.8 (5.0)
Sex				
Female	59 (77.6)	59 (75.6)	58 (75.3)	64 (82.1)
Male	17 (22.4)	19 (24.4)	19 (24.6)	14 (17.9)
Race				
Black or African American	16 (21.1)	10 (12.8)	9 (11.7)	13 (16.7)
White	57 (75.0)	66 (84.6)	67 (87.0)	65 (83.3)
Other^b^	3 (3.9)	2 (2.6)	1 (1.3)	0
Ethnicity				
Hispanic, Latino, or Spanish	3 (3.9)	3 (3.8)	0	1 (1.3)
Formal educational level				
High school graduate or less	9 (11.8)	8 (10.3)	12 (15.6)	9 (11.5)
Some college or technical	28 (36.8)	19 (24.4)	18 (23.4)	21 (26.9)
College graduate	39 (51.3)	51 (65.4)	47 (61.0)	48 (61.5)
No. living in household, mean (SD)	1.7 (0.9)	1.6 (0.6)	1.7 (0.9)	1.6 (0.9)
Participated during COVID-19 pandemic^c^	46 (60.5)	44 (56.4)	46 (60.5)	45 (57.7)
Health status				
Pain intensity, mean (SD)^d^	1.8 (2.0)	1.6 (2.0)	1.3 (1.9)	1.7 (1.9)
Interference from pain, mean (SD)^d^	0.9 (1.6)	1.0 (1.7)	0.9 (1.8)	1.1 (1.7)
Global physical health, mean (SD)^e^	39.1 (4.9)	39.5 (5.3)	40.0 (5.1)	39.2 (4.9)
Global mental health, mean (SD)^e^	51.8 (8.0)	52.3 (7.9)	52.9 (8.1)	50.8 (7.4)
Chronic conditions^f^				
Average No., mean (SD)	2.1 (1.2)	2.2 (1.1)	1.9 (1.0)	2.3 (1.2)
Cardiovascular	24 (31.6)	28 (35.9)	20 (20.6)	28 (35.9)
Diabetes	18 (23.7)	13 (16.7)	17 (22.1)	16 (20.5)
Lung	14 (18.4)	17 (21.8)	8 (10.4)	15 (19.2)
Arthritis	59 (77.6)	52 (66.7)	51 (66.2)	54 (69.2)
Osteoporosis	21 (27.6)	27 (35.1)	28 (36.8)	28 (36.4)
Self-reported PA levels consistent with guidelines^g^				
Aerobic >30 min per d	11 (14.5)	10 (12.8)	9 (11.7)	11 (14.1)
Strength training >2 times per wk	4 (5.3)	1 (1.3)	6 (7.8)	6 (7.7)
Balancing movements >3 times per wk	0	0	0	3 (3.9)
Objectively measured PA, mean (SD)				
Daily min of total PA^h^	185.9 (70.5)	167.7 (79.1)	177.9 (72.7)	177.9 (62.2)
Daily step count^h^	4387.6 (2295.2)	4012.0 (2437.2)	4314.8 (2430.1)	4197.5 (1890.7)
Daily min of MVPA^h^	23.2 (24.1)	21.7 (41.1)	20.9 (24.9)	19.4 (24.2)
Self-reported PA, mean (SD)^i^	110.6 (44.0)	108.3 (66.6)	101.8 (48.5)	102.2 (46.4)

Abbreviation: MVPA, moderate and vigorously intense physical activity (PA; aerobic movement fast and strenuous enough to burn off 3 to 6 times as much energy per minute than when sitting quietly and vigorous aerobic movement fast and strenuous enough to burn off ≥6 times as much energy per minute than when sitting quietly).

^a^ All intervention conditions included the Otago Exercise Program and a wearable PA monitor as core intervention components.

^b^ Other race includes Asian Indian, Chinese, Filipino, Indigenous, or some other race.

^c^ Participation in Ready Steady 3.0 was, at least in part, during the first phase of the COVID-19 pandemic, between March 2020 and May 2021.

^d^ Based on the Brief Pain Inventory–Short Form, a 9-item, self-report questionnaire used to evaluate the average intensity and impact of a person’s pain; scores range from 0 to 10, with higher scores indicating greater pain severity.^52^

^e^ Based on the Patient-Reported Outcomes Measurement Information System scale, version 1.1, Global Health, which is composed of standardized scores based on the US adult population, including older adults, with mean (SD) scores of 50 (10) and scores greater than 50 representing greater self-ratings of physical or mental health.^53^

^f^ Chronic conditions were self-reported.

^g^ Participants meeting each type of PA recommended in the PA guidelines as reported during screening.^3^ No enrollees reported meeting more than the minimum recommendations for more than 1 type of recommended PA: aerobic, strength training, and balance-challenging movements.

^h^ Mean (SD) daily minutes of total PA, daily step count, and daily minutes of MVPA were measured via triaxial accelerometers in wearable activity monitors, in which the algorithm to classify each minute as being in sedentary, light, moderate, or vigorous aerobic activity for each minute is proprietary.

^i^ Based on the self-reported PA Scale for the Elderly, in which scores range from 0 to more than 400, with higher scores indicating greater PA.^47^

### Primary Outcomes

**Figure 2.**
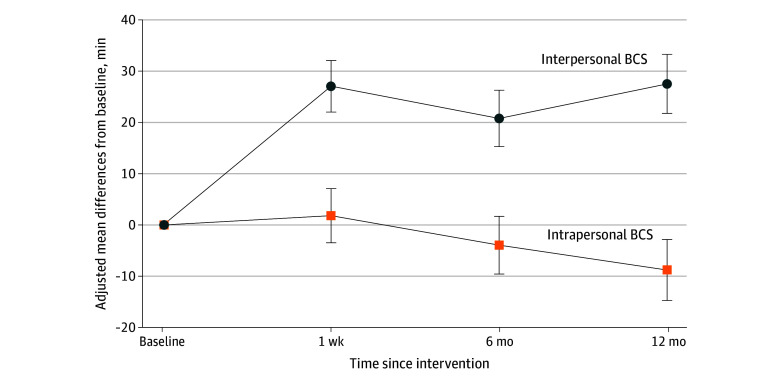
Effect of Intrapersonal and Interpersonal Behavior Change Strategies (BCSs) on Older Adults’ Total Physical Activity (PA) Quantity of PA was operationalized as mean (SE) changes in daily minutes of total PA (light, moderate, and vigorous intensities) averaged over 7 to 10 days. Physical activity was measured objectively using accelerometers within participants’ wearable activity monitors at 4 time points. Participants received 5 intrapersonal or interpersonal BCSs or did not (yes or no).

Table 2 summarizes unadjusted means (SD) for all outcomes. Figure 2 shows differences in the mean (SE) changes in daily minutes of total PA averaged over 7 to 10 days, measured using wearable PAMs at baseline and at each postintervention assessment.

**Table 2.** Primary and Ad Hoc Outcomes by Condition

Outcome	Intervention condition, unadjusted mean (SD)^a^			
	Intrapersonal behavior change strategies (n = 76)	Interpersonal behavior change strategies (n – 78)	Intrapersonal and interpersonal behavior change strategies (n = 77)	Attention control information about health and age (n = 78)
Daily total PA, min^b^				
Baseline	185.9 (70.5)	167.7 (79.1)	177.9 (72.7)	177.9 (62.2)
Time after the intervention				
1 wk	181.0 (68.2)	193.1 (82.1)	209.1 (79.3)	180.5 (68.5)
6 mo	175.1 (68.9)	186.7 (84.0)	198.0 (78.0)	180.0 (73.8)
12 mo	167.7 (68.6)	190.3 (99.1)	192.9 (80.4)	174.3 (72.0)
Daily step count^b^				
Baseline	4387.6 (2295.2)	4012.0 (2437.2)	4314.8 (2430.1)	4197.5 (1890.7)
Time after the intervention				
1 wk	4171.5 (1993.0)	4991.2 (2845.7)	5465.1 (2726.8)	4263.3 (2012.0)
6 mo	4053.1 (2058.7)	4683.7 (2940.5)	5087.4 (2959.0)	4206.6 (2184.1)
12 mo	3774.0 (2188.8)	4736.0 (3357.2)	4854.9 (2671.2)	4056.8 (2244.2)
Daily MVPA, min^b^				
Baseline	23.2 (24.1)	21.7 (41.1)	20.9 (24.9)	19.4 (24.2)
Time after the intervention				
1 wk	22.1 (28.6)	30.8 (35.3)	31.5 (33.2)	20.9 (26.1)
6 mo	19.7 (19.6)	29.1 (37.1)	29.8 (28.6)	23.1 (26.0)
12 mo	21.1 (25.9)	38.5 (52.4)	30.3 (30.6)	23.3 (26.4)
**Self-reported PA^c^**				
Baseline	110.6 (44.0)	108.3 (66.6)	101.8 (48.5)	102.2 (46.4)
Time after the intervention				
1 wk	120.7 (56.2)	119.2 (69.9)	121.1 (58.7)	111.1 (47.3)
6 mo	119.5 (65.8)	118.2 (65.1)	112.2 (60.1)	106.7 (53.0)
12 mo	123.0 (92.3)	113.8 (66.8)	112.4 (60.9)	107.1 (60.0)

Abbreviation: MVPA, moderate and vigorously intense physical activity (PA; aerobic movement fast and strenuous enough to burn off 3 to 6 times as much energy per minute than when sitting quietly and vigorous aerobic movement fast and strenuous enough to burn off ≥6 times as much energy per minute than when sitting quietly).

^a^ All intervention conditions included the Otago Exercise Program and a wearable physical activity monitor as core intervention components.

^b^ Measured using an accelerometer within participants’ commercially available, wearable PA monitor. All daily unadjusted means were an estimated time point, based on 7 to 10 days of data.

^c^ Measured using the self-reported PA Scale for the Elderly, in which scores range from 0 to more than 400, with higher scores indicating greater PA.^47^

After adjustment for baseline mean (SE) daily minutes of total PA, participants who received the intervention component with interpersonal BCSs (conditions 2 and 3 [n = 155]) exhibited greater increases in their PA than those who did not receive this component (conditions 1 and 4 [n = 154]) at 1 week (204 vs 177 PA minutes per day; adjusted difference, 27.1 [95% CI, 17.2-37.0]; *P* < .001), 6 months (195 vs 175 PA minutes per day; adjusted difference, 20.8 [95% CI, 10.0-31.6]; *P* < .001), and 12 months (195 vs 168 PA minutes per day; adjusted difference, 27.5 [95% CI, 16.2-38.8]; *P* < .001) after the intervention.

After adjustment for baseline minutes of PA, participants who received the intervention component with intrapersonal BCSs (conditions 1 and 3 [n = 153]) exhibited no significant changes in their PA relative to those who did not receive this component (conditions 2 and 4 [n = 156]) at time points 1 week: 192 vs 190 PA minutes per day (adjusted difference, 1.8 [95% CI, −8.6 to 12.2]; *P* = .73), 6 months: 183 vs 187 PA minutes per day (adjusted difference, −3.9 [95% CI, −15.0 to 7.1]; *P* = .49), and 12 months: 177 vs 186 PA minutes per day (adjusted difference, −8.8 [95% CI, −20.5 to 2.9]; *P* = .14) after the intervention.

The interaction effect of receiving interpersonal and intrapersonal components, adjusting for the other, on PA was not statistically significant at any postintervention time point (eTable 1 in Supplement 2). Self-reported PA did not differ significantly by receipt of the intervention components with intrapersonal BCSs (no vs yes) or with interpersonal BCSs (no vs yes) nor was there a significant interaction between these components at any postintervention time point (eTable 1 in Supplement 2).

### Post Hoc Analyses of Daily Step Counts and MVPA

After adjustment for baseline mean (SE) daily step counts, participants who received the intervention component with interpersonal BCSs exhibited greater increases in their mean (SE) daily step counts than those who did not receive the component at each postintervention time point: 1 week (5251 vs 4193 steps per day; adjusted difference, 1058 steps per day [95% CI, 796.2-1320.7]; *P* < .001), 6 months (4894 vs 4118 steps per day; adjusted difference, 776 steps per day [95% CI, 485.8-1065.9]; *P* < .001), and 12 months (4819 vs 3894 steps per day; adjusted difference, 926 steps per day [95% CI, 566.3-1285.1]; *P* < .001).

Receipt of the intervention component with interpersonal BCSs also elicited a significant increase in mean (SE) daily minutes of MVPA at all 3 postintervention assessments: 1 week (31.0 vs 21.7 MVPA minutes per day; adjusted difference, 9.3 [95% CI, 3.7-14.9]; *P* < .001), 6 months (29.2 vs 21.6 MVPA minutes per day; adjusted difference, 7.6 [95% CI, 2.1-13.1]; *P* = .007), and 12 months (34.1 vs 22.5 MVPA minutes per day; adjusted difference, 11.6 [95% CI, 5.5-17.7]; *P* < .001).

Analyses indicated that receipt of the intrapersonal component had no significant effect on mean (SE) daily step counts or mean (SE) daily minutes of MVPA. There were not any significant interaction effects of receiving intrapersonal and interpersonal components, adjusting for the other, on these metrics at any postintervention time point (eTable 1 in Supplement 2).

## Discussion

To our knowledge, the RS 3.0 randomized clinical trial is one of the first studies to test the distinct and combined effects of more than 1 type of BCS^6^ within a PA intervention on older adults’ total PA up to 12 months after an intervention.^21^ In a sample of community-dwelling older adults with low baseline PA, interventions that included interpersonal BCSs led to significant initial and sustained increases in objectively measured total PA and MVPA.

While this finding is generally consistent with prior research on PA interventions for older adults, it advanced this literature by specifically identifying interpersonal BCSs within PA interventions as helpful in promoting sustained PA in older adults.^54^ Overall, the magnitude of the effect of PA interventions with interpersonal BCSs on participants’ total PA and MVPA was clinically meaningful^55^ and exceeded short-term outcome effectiveness benchmarks recently published.^56^ The findings also replicated a prior study, RS 2.0, by some of us,^23^ in a larger sample and longer follow-up. Other intervention studies with interpersonal BCSs plus intrapersonal BCSs have also shown increases in PA of older adults,^57^ African American adult women,^58^ and adolescents.^59^ However, more specific evidence about the unique effects of intrapersonally vs interpersonally oriented BCSs is sparse and inconclusive.^8,9,14^ It is possible that the peer-to-peer sharing and learning,^13^ undergirding discussions about interpersonal BCSs in RS 3.0, supported the development of social capital^18^ (eg, visiting with neighbors, attending organized group meetings, and networks) and social integration (eg, involvement with peripheral social ties).^60^

Evidence generated by RS 3.0 regarding the importance of integrating interpersonal BCSs into PA interventions for older adults is promising and has implications for future research. The effectiveness of this approach, as well as potential barriers and enablers to its dissemination and implementation, needs to be investigated across diverse settings.^61,62,63^ Examining the feasibility and benefit of integrating the intervention component of RS 3.0 comprising interpersonal BCSs into existing programs that promote the uptake and maintenance of PA among older adults (eg, Active Living Every Day, Walk With Ease)^64^ is also warranted. Additional research is also needed to identify the mechanisms through which interpersonal BCSs affect PA while also accounting for the upstream conditions and contexts (eg, social determinants of health) in which they operate to ensure that existing inequities are not widened but are reduced.^6,61,65,66,67^

The observation that using intrapersonal BCSs did not distinctly elicit increases in PA in older adults would appear to contradict the general conclusions drawn in some literature reviews that interventions with individual-level cognitive and behavioral strategies are associated with greater increases in PA.^19,54^ However, more specific findings from some reviews indicate that certain BCSs, such as goal setting and action planning, are not associated with increased PA. In contrast, others are, such as coping planning.^8,9^ Given that most studies included in these reviews have evaluated intrapersonal BCSs bundled together with all intervention content (eg, interpersonal BCSs) and delivery components, it is difficult to discern which BCSs and components contributed to positive effects and which did not.^8,15,19^ Future research is needed to identify whether there are specific intrapersonal BCSs that are worth including in PA interventions for older adults.^3^

Findings of no significant intervention effects on self-reported PA measured are consistent with past research that shows that intervention effects are greater among studies using objective measures than those using self-report measures.^49^ Well-known biases associated with self-report measures of PA,^68^ as well as patterns of inconsistencies shown in prior research (eg, those with very low PA self-report more activity than measured using an accelerometer),^69^ could have contributed to these findings. Indeed, it has been shown that while correlations between self-report and objectively measured PA in older adults are positive, the strength of these correlations tends to be weak or moderate.^70,71,72^ Thus, future research should use objective measures when possible to estimate PA quantity and use self-report measures for other reasons, such as to explore perceived compared with actual PA and the types, domains, and contexts of one’s PA.^49,70^ Future publications will focus on secondary and exploratory outcomes measured in this study, including fall rates, quality of life, and putative physical and psychosocial mechanisms.^24^

### Limitations

This study has limitations. Wearable PAMs, included as a core intervention component, may not be widely accessible. Although several baseline characteristics of the sample in our study represent older adults in Minneapolis and Saint Paul, the study sample was not large enough to examine intervention effects in subgroups of participants (eg, varied levels of baseline PA, disabilities, or chronic conditions).^65^ A well-known limitation of total PA minutes, step count, and MVPA metrics is that they may not fully capture all PA types recommended in the guidelines (eg, balance challenging and leg strengthening) or some aerobic PAs (eg, bicycling and swimming).^3^ Finally, conducting the study during the COVID-19 pandemic may have influenced PA for participants in dynamically varied ways. However, the number of participants enrolled before or after the start of the COVID-19 pandemic was similar across all 4 conditions (Table 1).

## Conclusions

In this randomized clinical trial among community-dwelling older adults with low levels of PA, an 8-week intervention comprising an evidence-based PA protocol, a PAM, and interpersonal BCSs involving peer-to-peer learning and sharing, but not intrapersonal BCSs, resulted in significant increases in total PA and MVPA, which were sustained for up to 12 month after the intervention. Future research should examine approaches to disseminating and implementing the RS intervention and its interpersonal BCS component within existing community-based programs and services.

## Notes

Supplement 1.
Trial Protocol

Supplement 2. **eTable 1.** Baseline Value Adjusted 2 (Intrapersonal: Yes, No) x 2 (Interpersonal: Yes, No) ANCOVA Results for Change in Physical Activity Outcomes Postintervention at 1 Week, 6 Months, and 12 Months **eTable 2.** Comparison of Baseline Characteristics and Physical Activity Outcome Values Among All Participants (Overall), Those Who Were Randomized With a Partner, and Those Who Were Not Randomized With a Partner **eFigure.** Longitudinal Analysis of Effects of Interpersonal Behavior Change Strategies on Primary Outcome of Average Daily Minutes of Total Physical Activity **eTable 3.** Potential Clustering Effects
eReferences

Supplement 3.
Data Sharing Statement
