# Supplementary material for: Effect of Intrapersonal and Interpersonal Behavior Change Strategies on Physical Activity Among Older Adults: A Randomized Clinical Trial
Source: JAMA Netw Open. 2024 Feb 29;7(2):e240298. doi: 10.1001/jamanetworkopen.2024.0298 (PMC10905305; doi:10.1001/jamanetworkopen.2024.0298)
Supplement: Supplement 1. — Trial Protocol [file jamanetwopen-e240298-s001.pdf]

Supplemental Online Content
Study Protocol

Effects of intrapersonal and interpersonal behavior change strategies on older adults' physical activity 1-week to 1-year post-intervention: A randomized factorial trial

Table of Contents

Contents

CHAPTER 1- OBJECTIVES **7**

1.1: PURPOSE *7*

CHAPTER 2 - BACKGROUND **8**

2.1 SIGNIFICANCE OF RESEARCH QUESTION/PURPOSE *8*

2.2 EXISTING LITERATURE *8*

CHAPTER 3 - STUDY OUTCOMES **11**

3.1 PRIMARY OUTCOME *11*

3.2 SECONDARY OUTCOME(S) *12*

3.3: EXPLORATORY OUTCOME(S) *12*

CHAPTER 4 - STUDY INTERVENTION **12**

4.1: DESCRIPTION *12*

CHAPTER 5 – PROCEDURES INVOLVED **15**

5.1 STUDY DESIGN *15*

5.2 STUDY PROCEDURES *15*

5.4 FOLLOW-UP *19*

CHAPTER 6 – DATA BANKING **19**

CHAPTER 7 – SHARING OF RESULTS WITH PARTICIPANTS **19**

7.1 DISSEMINATION *19*

CHAPTER 8 – STUDY DURATION **19**

CHAPTER 9 – STUDY POPULATION **19**

9.1 INCLUSION CRITERIA *19*

9.2 EXCLUSION CRITERIA *20*

9.3 SCREENING *20*

CHAPTER 10 – VULNERABLE POPULATIONS **21**

CHAPTER 11 – NUMBER OF PARTICIPANTS **21**

11.1: NUMBER OF PARTICIPANTS TO BE CONSENTED *21*

CHAPTER 12 – RECRUITMENT METHODS **21**

12.1 RECRUITMENT PROCESS *21*

12.2: SOURCE OF PARTICIPANTS *22*
12.3: IDENTIFICATION OF POTENTIAL PARTICIPANTS *22*
12.4: RECRUITMENT MATERIALS *22*
12.5: PAYMENT *22*

CHAPTER 13 – WITHDRAWAL OF PARTICIPANTS 23
13.1: WITHDRAWAL CIRCUMSTANCES *23*
13.2: WITHDRAWAL PROCEDURES *23*
13.3: TERMINATION PROCEDURES *23*

CHAPTER 14 – RISKS TO PARTICIPANTS 23
14.1: FORESEEABLE RISKS *23*
14.2: REPRODUCTION RISKS *25*
14.3: RISKS TO OTHERS *25*

CHAPTER 15 – INCOMPLETE DISCLOSURE OR DECEPTION 25

CHAPTER 16 – POTENTIAL BENEFITS TO PARTICIPANTS 25
16.1: POTENTIAL BENEFITS *25*

CHAPTER 17 – DATA MANAGEMENT 25
17.1: DATA ANALYSIS PLAN *25*
17.2: POWER ANALYSIS *26*
17.3: DATA INTEGRITY *27*
17.4: DATA SECURITY *27*

CHAPTER 18 – PROVISIONS TO MONITOR THE DATA TO ENSURE THE SAFETY OF PARTICIPANTS 30
18.1: DATA INTEGRITY MONITORING *30*
18.2: DATA SAFETY MONITORING *30*

CHAPTER 19 – PROVISIONS TO PROTECT THE PRIVACY INTERESTS OF PARTICIPANTS 34
19.1: PROTECTING PRIVACY *34*
19.2: ACCESS TO PARTICIPANTS *35*

CHAPTER 20 – CONSENT PROCESS 35
20.1: CONSENT PROCESS (When consent will be obtained): *35*
20.2 COMPENSATION OF RESEARCH RELATED INJURY *36*
20.3 CONTRACT LANGUAGE *36*
20.4: WAIVER OR ALTERATION OF CONSENT PROCESS *36*
20.5: NON-ENGLISH SPEAKING PARTICIPANTS *36*

20.6: PARTICIPANTS WHO ARE NOT YET ADULTS 36
20.7: COGNITIVELY IMPAIRED ADULTS, OR ADULTS WITH FLUCTUATING OR DIMINISHED CAPACITY TO CONSENT 36
20.8: ADULTS UNABLE TO CONSENT 37
CHAPTER 21 – SETTING 37
21.1: INTERNATIONAL RESEARCH 37
21.2: COMMUNITY-BASED PARTICIPATORY RESEARCH 37
21.3: RESEARCH SITES 38
CHAPTER 22 – MULTI-SITE RESEARCH 39
CHAPTER 23 – REFERENCES 40

ABBREVIATIONS

ANCOVA	Analysis of Covariance
CDC	Centers for Disease Control and Prevention
EASY	Exercise and Screening for You
IRB	Institutional Review Board
HIPAA	Health Insurance Portability and Accountability Act
NIA	National Institute on Aging
PA	Physical Activities
PHI	Patient Health Information
QOL	Quality of Life
SPPB	Short Physical Performance Battery
WMT	Wellness Motivation Theory
REDCap	Research Electronic Data Capture
RAPA	Rapid Assessment of Physical Activity
RA	Research Assistant
PROMIS	Patient-Reported Outcomes Measurement Information System
UMN	University of Minnesota
NNINR	National Institute of Nursing Research
RE-AIM	Reach- Effectiveness –Adoption- Implementation-Maintenance
SAS	A statistical analysis software
SPSS	A statistical analysis software

STUDY SUMMARY

**Study Title**	Community-based intervention effects on older adults' physical activity & falls
**Study Design**	2 x 2 factorial experimental design
**Primary Objective**	Determine which experimental intervention component(s) increase physical activity (PA) among community-dwelling older adults post-intervention: immediately, 6 months, and 12 months
**Secondary Objective(s)**	Determine which experimental intervention components decrease fall occurrence and increase quality of life (QOL) among community-dwelling older adults 12 months post-intervention
**Primary Study Intervention or Interaction**	An 8 week PA intervention that includes the following components:<br>1. Two sets of behavioral strategies: interpersonal and intrapersonal<br>2. Otago PA protocol<br>3. PA-monitor (Fitbit Accelerometers)<br>4. Attention Control Content: Health & Age Topics from the National Institute on Aging (NIA)
**Study Population**	Older adults ≥ 70 years of age
**Sample Size (number of participants)**	N = 308
**Study Duration for Individual Participants**	15 to 18 months

CHAPTER 1- OBJECTIVES

1.1: PURPOSE

Purpose, specific aims, or objectives.

Our research team will conduct a 2 x 2 factorial experiment testing the individual and combined effects of two empirically and theoretically relevant sets of behavior change strategies on community-dwelling older adults' physical activity. To do this we will randomize participants $\geq$ 70 years old (n = 308) to 1 of 4 experimental conditions. All conditions include an evidence-based physical activity protocol endorsed by Centers for Disease Control and Prevention (CDC) for use by all older adults, including those with frailty and multiple co-morbidities and the commercially available physical activity monitor (e.g., Fitbit) to augment intervention delivery. Intervention components that are experimental and vary by condition are the sets of behavior change strategies which will be combined with the physical activity protocol and the physical activity monitor. Condition 1 has no specific behavior change strategies; Condition 2 includes an intervention component comprised of 5 interpersonal behavior change strategies, such as facilitating social support and social comparison; Condition 3 includes an intervention component comprised of 5 intrapersonal behavior change strategies, such as setting personally meaningful goals; and Condition 4 includes both sets of behavior change strategies -- 5 interpersonal strategies combined with 5 intrapersonal behavior change strategies.

To fully examine the effects of these experimental components, we have delineated Primary, Secondary and Exploratory Aims:

Primary Aim: Determine which experimental intervention component(s) increase PA among community-dwelling older adults post-intervention: immediately, 6 months, and 12 months. **Hypothesis:** Participants receiving the interpersonal set of behavior change strategies will have clinically meaningful increases in PA post- intervention (at all 3 time-points), compared to participants not receiving these strategies.

Secondary Aim: Determine which experimental intervention component(s) decrease fall occurrence and increase quality of life (QOL) among community-dwelling older adults 12 months post-intervention. **Hypotheses**: Participants receiving the set of interpersonal behavior change strategies will have clinically meaningful reductions in falls and increases in QOL, 12 months post-intervention, compared to participants not receiving these strategies.

Exploratory Aim: Evaluate experimental intervention component effects on targeted psychosocial constructs (social support; readiness; self-regulation) and physical constructs (functional leg strength and balance), which are theorized as mechanisms of change--and whether these mechanisms mediate the effects of experimental intervention components on PA and falls. **Hypotheses:** Receiving the interpersonal behavioral change strategies, relative to not receiving these strategies, will elicit increases in targeted psychosocial constructs and increases in physical constructs, which in turn will mediate the intervention's effects on PA behavior and falls.

CHAPTER 2 - BACKGROUND

2.1 SIGNIFICANCE OF RESEARCH QUESTION/PURPOSE

A description of the relevant prior research and gaps in current knowledge for your research question.

Every year, 1 in 3 people aged 65 and older falls; 25% of which result in serious injury or death and have devastating effects on QOL.[1, 5] Despite evidence that PA targeting leg strength and balance reduce falls, less than 12% of older adults engage in these activities on a regular basis and fall rates continue to increase.[7] Research examining intervention strategies that motivate older adults to engage in PA is scarce and inconclusive. There is a compelling need to identify specific behavioral change strategies that effectively and efficiently motivate older adults to sustain increased levels of PA.

Several behavioral change strategies have the potential to motivate older adults to engage in PA, such as facilitating social support;[19] barriers management;[27] goal-setting;[28] habit formation ;[29] and self-monitoring outcomes.[30] To date, intervention studies typically combine 7-10 strategies and evaluate them as part of an "intervention package" compared to a control group. Thus, it is not known which strategy, or set thereof, are effective and should be included in efficient and translatable interventions. The **objective of this proposal** is to **identify behavioral change strategies that elicit increased PA, sustained for 12 months and, in turn, lead to a reduction in falls and improved QOL**. Building on prior research, the proposed study will use a factorial experimental design to test the relative influence of behavioral change strategies separated into two components. These components are comprised of distinct sets of behavioral change strategies that represent two different ways of motivating people to take action: interpersonal and intrapersonal, consistent with theories of behavioral change[16, 22] and lifespan development, [46] as well as empirical evidence.[33] The components will be combined with core intervention content that includes Otago, [10] an evidence-based fall- reducing PA protocol, and a PA-monitor for self-tracking.

2.2 EXISTING LITERATURE

Scientific background for, rationale for, and significance of the research based on the existing literature and how will it add to existing knowledge.

Fall rates continue to increase and PA rates remain low among older adults. The complex public health problems of falls and inactivity create significant social and economic burdens. Every year, 1 in 3 people aged 65 and older falls; 25% result in serious injury or death.[1] Injurious and fatal fall rates increase exponentially with age, particularly after age 70.[1] Serious injuries caused by falls, such as traumatic brain injuries and fractures, [2] often require intense healthcare services within emergency rooms, hospitals, and nursing homes.[3] They also increase fear of falling, [4] decrease mobility, and decrease QOL.5 The direct healthcare costs of fall-related injuries in older adults are $34 billion annually.[1] Decreased leg strength, poor balance, and altered gaits are the most common causes of falls. These causes can be mitigated with the regular practice of leg strengthening and balance-challenging activities.[6] Walking and flexibility movements augment these activities. Previous research demonstrates that approximately 35% of injurious/fatal falls can be prevented with fall-reducing PA,[6] and that PA is associated with improved QOL.[5] Despite public health efforts to disseminate and implement this

evidence, less than 12% of people aged 65+ engage in PA as recommended [7] and fall rates continue to rise. Thus, knowledge of fall-reducing PA has not translated to increased PA rates in the community of older adults, or made an impact on falls. *It is essential to augment what is known; the types and doses of PA that reduce falls, with what is not known; intervention strategies that motivate older adults to sustain increased PA*.[8]

Fall-reducing PA interventions. In a Cochrane review, Gillespie and colleagues identify fall-reducing PA among the most effective fall prevention interventions, yet few studies describe or evaluate strategies to increase "uptake" of and "adherence" to fall-reducing PA.[6] The dozens of fall-reducing PA intervention studies conducted over the last several decades have generated substantial knowledge about which PAs work to reduce falls and injuries among older adults, including those with multiple chronic conditions, pain, and frailty.[9] Meta-analyses show the effects of fall-reducing PA interventions on fall rate ratios range from 0.56 to 0.82.[6,10,11] One example of an evidence-based program emerging from this literature is the Otago protocol, which is cost-effective when delivered 1:1 in home settings [12] and when delivered in small groups.[13] Otago and other similar PA protocols include instruction and supervisory strategies, but not content to increase the initiation or maintenance of PA. The few intervention studies that have included such content show conflicting results due, in part, to the use of self-report PA measures.[9,14] *Future studies need to build upon existing evidence-based, fall-reducing PA protocols by combining them with evidence-based strategies to motivate older adults' PA, and estimate their effects on objectively measured PA (e.g., accelerometer)*. Below we identify fundamental building blocks that inform such progress.

Motivation for fall-reducing PA. Motivation, simply defined as why one moves to action, is a key determinant of older adults' PA and a dynamic process dependent on key constructs.[15-18] For instance, social support for PA from important others (e.g., friends, neighbors, family, providers) is known to influence PA engagement.[19,20] Readiness for PA refers to the identification of goals consistent with personal values and health-related behavior, as well as commitment and confidence for attaining those goals.[21,22] Self-regulation refers to self-monitoring progress toward goal attainment,[23] identifying discrepancies between what is desired and what is achieved, and adjusting plans accordingly.[24] Together, as postulated by the wellness motivation theory (WMT),[25] and consistent with some research,[26] these constructs influence the maintenance of personally valued health behaviors, such as PA. Despite strong evidence and theoretical explanations of motivation as a PA determinant, there is a dearth of fall-reducing PA research that evaluates motivational constructs.[6,9] *Motivational constructs, based on empirical evidence and theory, can be targeted in new multi- component fall-reducing PA interventions, using behavior change strategies*.

Which behavioral change strategies have potential to motivate older adults to sustain increased PA? Prior research, in the field of PA, indicates that many behavioral change strategies target motivational constructs and have the potential to increase older adults' PA, such as facilitating social support; [19] barriers management; [27] goal-setting; [28] habit formation; [29] and self-monitoring outcomes.[30] To date, intervention studies have typically included 7-10 strategies and evaluate them as part of an "intervention package" compared to control groups. To address questions about which of these strategies have an impact on key outcomes, meta-analyses have been conducted to examine effects of individual and sets of similar behavioral change strategies, but show mixed results due to difficulties disentangling intervention content post- experiment.[31,32] The studies do suggest that different strategies, or sets of strategies, may benefit older adults more than younger adults,[33] and may result in sustained increases in PA.[32] A weakness of prior research is that it has not produced sufficient evidence to understand which

strategies should, or should not, be included in streamlined multicomponent fall-reducing interventions. *A strength of prior research is that it highlights important methodological strategies that can be used in future research to improve reproducibility and scientific rigor, such as a) clearly describing each strategy used and how it is operationalized; and b) separating behavioral change strategies into distinct intervention components based on theoretical, empirical, and practical rationale, so that the contributions of each component may be examined experimentally*.

Questions about which behavioral change strategies motivate older adults to sustain increased fall-reducing PA warrant factorial experimental design. Factorial experiments are a practical approach to optimizing interventions for effectiveness, efficacy, and translatability through the identification of intervention components that meaningfully contribute to overall intervention effects. Components refer to distinct intervention features related to content (e.g., sets of behavioral change strategies), delivery, or other logistics. Other experimental designs, such as multiple arm comparative experiments and two arm randomized controlled trials, cannot directly address specific questions about the individual and combined effects of intervention components, and thus would be inefficient in the context of investigating their relative contributions.[35] *Factorial experiments are the best choice for future research that investigates combining fall-reducing PA interventions with behavioral change strategies, separated into distinct components, because these experiments enable answering questions about the individual and combined effects of intervention component(s) and thus also optimize the intervention for efficiency*.

PA-monitors can facilitate intervention delivery and data collection. Numerous commercially available PA-monitors (e.g., Fitbit) are affordable, acceptable and useful to older adults, [36] and have the potential to facilitate PA.[37] Additionally, recent evidence suggests that PA-monitors, Fitbit OneTM in particular, provide accurate measurement of steps, PA duration and intensity.[38-41] There are no fall-reducing PA intervention studies that we are aware of, with the exception of our preliminary studies that describe integrating PA-monitors. *Evidence about commercially available PA-monitors can be used to inform intervention design and measurement methods*.

Community-based fall-reducing PA interventions have the potential to improve PA promotion efforts. Prior research suggests that community engaged research yields more meaningful collaborations; improved interventions that are more sustainable; and improved health outcomes on both individual and community levels.[42] *Fall-reducing PA interventions that are community-based provide a means for developing research-community partnerships, as well as interventions with potential to be more sustainable*.

In sum, this proposal addresses two serious, common, and preventable public health problems: falls and low PA, by integrating strengths and limitations of prior research. The intervention combines an evidence-based fall-reducing PA protocol targeting leg strength and balance; plus key behavioral change strategies targeting motivational constructs. These key behavioral change strategies, which are empirically based and theoretically informed, are separated into two intervention components comprised of distinct sets of intervention strategies: interpersonal (e.g., fostering social support for PA; facilitating friendly social comparison) and intrapersonal (e.g., facilitating the development of goals and plans). The individual and combined effects of these components on long-term outcomes, and motivational constructs, will be tested using a factorial experiment. Commercially available PA-monitors will be integrated into the intervention and the accelerometers built in to the monitors will be used to objectively

measure PA. The successful implementation of this proposal relies on partnerships with community stakeholders, which our team has established over the last three years. This proposal's research will optimize a multicomponent intervention whose behavioral change strategies produce meaningfully sustained effects on increased PA, reduced falls, and improved QOL. *This contribution will be significant because it is expected to foster the integration of biomechanically-oriented fall-reducing PA protocols with behavioral change strategies that impact health outcomes*.

Innovation. The design of this proposal's study, and methods used to achieve its aims, are highly innovative. The design, driven by the inter-related problems of falls and low PA rates in older adults, engages the science of behavior change and gerontology to identify and test strategies that potentially motivate sustained PA, and in turn reduce falls and improve QOL. As illustrated in **Figure 2**, the conceptual model for this intervention includes critical elements that guide the serial assessment of mechanisms and outcomes, which will enable us to observe and describe intervention effects, as well as patterns of change over time. The design of this proposal is consistent with systematic approaches to optimizing behavioral change interventions that increase understanding of what works, when, and how; and that lead to effective, efficient, and translatable programs.[43,44] Such approaches are a novel enhancement to existing fall-reducing PA protocols. Factorial experimental methods used within this proposal complement the design described here and allow us to directly test each aim's working hypotheses.[35] *The proposed research is innovative, in our opinion, because it represents a substantive expansion of biomechanically-oriented fall-reducing PA protocols to a) examine which behavioral change strategies elicit sustained effects (e.g., 12 months) on increased PA, as well as on falls, and QOL; and b) explore underlying mechanisms of PA and falls*. Successful completion of this research is expected to strengthen PA promotion efforts in older adult populations by identifying strategies that support older adults to autonomously sustain increased PA, and by gaining an understanding of mechanisms that enable the application of our study results to other areas of health.[45]

Figure 2. Intervention Conceptual Model

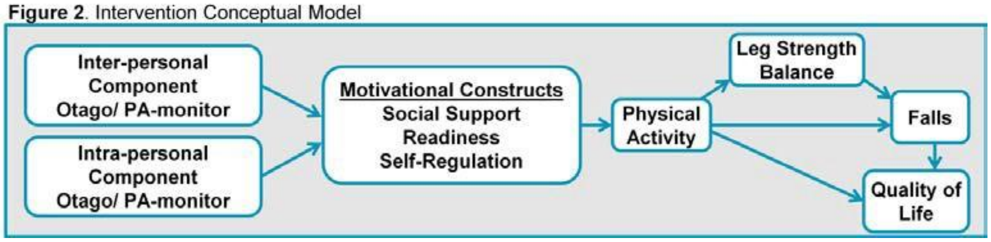

CHAPTER 3 - STUDY OUTCOMES

3.1 PRIMARY OUTCOME

The primary study endpoint, event, or outcome we will be evaluating or observing.

The **primary outcome** is the quantity of PA: the average minutes of total PA per week (light, moderate and vigorous intensities) measured objectively and via self-report.

3.2 SECONDARY OUTCOME(S)

Secondary study endpoints, events, or outcomes you will be evaluating or observing.

Secondary outcomes include:

- Fall rates
- Quality of life

3.3: EXPLORATORY OUTCOME(S)

Other exploratory variables include:

- Functional strength and balance
- Social resources
- Readiness
- Self-regulation
- Pain (intensity and impact on function)
- Medication inventory
- Sex as a biological variable
- Covid 19 (impact of mitigation/ containment efforts on health and physical activity)

CHAPTER 4 - STUDY INTERVENTION

4.1: DESCRIPTION

Intervention description.

Dose and delivery. We carefully considered the dose of 8 weekly contacts, each lasting 90 minutes, based on triangulated data from our prior research and feedback from community stakeholders. Our prior research [9] and the CDC compendium of fall prevention interventions 61 describe a broad range of doses that elicit promising effects with 8 weeks-12 contact hours on the low side of the range and 52 weeks-104 contact hours on the high side of the range. Also, our preliminary studies showed intervention session attendance was good across conditions and in post-intervention interviews, participants indicated this dose was acceptable: some even suggested we increase intervention duration to 12 weeks. Community stakeholders including older adults, center leaders, and healthcare providers advised us to increase the amount of time spent during contacts from 60 minutes to 90 minutes to ensure participants did not feel "rushed." They also advised us to conduct intervention and data collection meetings between 9 am and 4 pm, and to limit group size to 4-6.

Adjustments for Coronavirus: March 17, 2020

Intervention visits in this study are done in small group (4 to 6 people) gatherings during which we practice the Otago Exercise Program movements together, and a coach facilitates discussions focused on behavior change strategies being tested. We enroll one cohort of 16 to 24 participants at a time, so they can complete the 8-week course. In

light of the current situation, we will delay future cohorts until we are able to deliver the small group intervention in person again.

However, our current cohort (11 of 13) is in week 6 of the 8-week intervention. Thus, we plan to conduct these by phone conferencing using UMN Zoom. We will not use the video portion of Zoom because not all participants have access to the internet. We will conduct each meeting according to our intervention manual, which delineates topics for discussion and exercise per Otago protocol. Similar to in-person intervention meetings and when participants perform these exercises at home independently (2 times per week to every day), there is a small risk of discomfort or injury. Consistent with our protocol and the Otago Exercise Program manual we will minimize the risk using several strategies. First, the exercises in Otago are considered safe for older adults, including those with frailty and multiple chronic conditions. Second, we will ensure that all phone conference participants have water, and are near sturdy structures during the exercises, such as a non-rolling chair or a counter. Third, we will remind participants to stop exercising if they feel discomforts such as joint pain or shortness of breath. Finally, at this point in the intervention, all participants who will participate in the conference call have mastered the exercises and know how to personally adapt them according to ability, comfort, and preferences.

Table 3 highlights condition and content by time. Each contact is 90 minutes: 10 for questions and answers; 40 for Otago PA; 20 for interpersonal behavior change strategies; 20 for intrapersonal behavioral change strategies; and, in lieu of behavioral change strategies--20-40 for attention control topics related to Health & Age, based on the National Institute of Aging website, Health & Aging.[62]

Table 3. Condition, Content, and Time

Time in Minutes	Condition and Content			
	Otago/PA	Otago/ PA + Inter-personal	Otago/ PA + Intra-personal	Otago/ PA + Inter-personal + Intra-personal
10	Q& A	Q&A	Q&A	Q&A
40	Otago	Otago	Otago	Otago
20	Health & Age	Inter-personal	Health & Age	Inter-personal
20	Health & Age	Health &Age	Intra-personal	Intra-personal

The social milieu in each small group meeting will support friendly and positive interactions and discussions guided by curricula topics and objectives. All content (experimental, attention control, core) is summarized in the next six paragraphs.

Experimental component: Interpersonal content. In the interpersonal component, a dialogue between small group participants will be facilitated focusing on their knowledge and experiences integrating fall-reducing PA into their social routines, providing and receiving social support for exercise, and using relevant community resources. Non-competitive, friendly, social comparison and exchange will also be facilitated, focusing on sharing different ways to practice fall-reducing PA; PA patterns derived from personal PA-monitor data; what others think about PA; and sharing PA data with important others (e.g., family, friends, providers).

Experimental component: Intrapersonal content. The intrapersonal component discussions will focus on guiding participants to develop, and modify as needed, personally meaningful goals and plans, using the National Institute on Aging's Go4Life[63] goal- setting and weekly planning PA worksheets. Discussions will also address personal barriers management; habit formation; coping with potential setbacks; and self-monitoring PA outcomes, congruent with personal goals.

Attention control content: Health & Age. Similar to our preliminary research, we will provide attention control topics about Health & Age, from the National Institute of Aging website including information about falls, pain, nutritional supplements, sleep, hearing, memory, and vaccinations.

Core content: Otago (PA protocol). The evidence-based Otago protocol guides the core fall-reducing PA content in this study. It is feasible and effective in community-dwelling older adults, including those with multiple chronic conditions (e.g., osteoporosis), pain, and frailty.[10, 13, 64] Our team and others have successfully implemented the Otago PA protocol in small groups.[13, 49] The overarching goal of Otago in this study is to facilitate participants' mastery and individualization of specific movements and exercises so they can self-direct their practice of these outside the small group setting. The program includes 5 flexibility movements (back extension and ankle, head, neck, trunk movements), 5 leg strengthening exercises (knee extensor, knee flexor, hip abductor, ankle plantar flexes, ankle dorsiflexes), 12 balance movements (knee bends, backwards walking, walking and turning, sideways walking, tandem stance, tandem walk, one leg stand, heel walking, toe walking, heel-toe walking backwards, sit to stand, stair walking), and a walking plan. The number, intensity and duration of movements are gradually increased.[65] Thus, time spent on the combined movements and exercises slowly increase across the 8-week intervention from approximately 15 to 35 minutes per meeting. We encourage participants to increase their walking time by 20% of their baseline average -up to 30 minutes per day. We also encourage participants to self-direct their practice of Otago PA that they have mastered, outside the small group meetings. Some choose to practice their leg-strengthening and balance PA during one timeframe. Others choose to practice different types of PA at different times of the day (e.g., balance exercises while waiting for coffee to brew, leg-strengthening exercises while watching TV). Similarly, some choose to take 30-minute walks once per day, others prefer to take break their walking time up into 10 minute bouts. In total, participants are encouraged to practice their personalized Otago PA protocol every other day (approximately 70 minutes per week) and to walk approximately 30 minutes per day (150 minutes per week), which is consistent national and international PA guideline recommendations for minimal PA among older adults, including those with multiple chronic conditions.[59] When participants cannot practice the amounts of PA recommended in Otago due to health conditions, they are encouraged to be as physically active as their abilities and conditions allow.[59,66]

Core content: PA-monitor. We will use Fitbit, a commercially available PA-monitor. Matt Buman, Ph.D. (Consultant) is an expert in mobile health who will advise our research team about the model of Fitbit and provide input about managing and analyzing accelerometer data, which we accomplished in the past using Fitabase.[67] Features of PA-monitors that were important to participants in our preliminary studies include displays with real-time feedback in the form of actual steps and distance, as well as positive messages that encourage about PA.[36,50] Features essential for researchers include built-in accelerometers that accurately measure steps and PA duration. Each device will be set up and programmed prior to distributing. We will assign a unique monitor identification number to each monitor; not related to participant's study ID or personally identifying information. Also, we will program monitor parameters (e.g., step goals) to minimal levels. During the baseline data collection meeting, we will provide each participant with a PA-monitor and a 15-20-minute orientation to review and demonstrate its basic functions: wearing, charging, and reading the display. Participants will also have the opportunity to return demonstrate. In addition, RAs will be available to help participants troubleshoot, as needed, via telephone and in person, after intervention meetings.

Intervention meetings include discussions about the PA-monitors, but topics vary per assigned condition (see **Table 4**) above.

CHAPTER 5 – PROCEDURES INVOLVED

5.1 STUDY DESIGN

Experimental. We will address this proposal's aims and hypotheses using a 2x2 factorial experiment where one factor represents receipt of an interpersonal intervention component (No vs Yes) and the second factor represents receipt of an intrapersonal intervention component (No vs Yes). Three hundred and eight community-dwelling older adults will be randomized to 1 of 4 conditions, which represent distinct combinations of experimental components (sets of interpersonal and intrapersonal behavior change strategies) and core intervention content (evidence based physical activity protocol and a physical activity monitor). The study will be implemented in 13 waves over 48 months, within Minneapolis and St. Paul community centers. Interventions for each condition will be delivered to small groups of participants (4-6) randomized to the same condition. These small groups will meet every week for approximately 90 minutes over the course of 8 weeks.

5.2 STUDY PROCEDURES

Figure 3 outlines an overview of participant activities in this study. Participants at least 70 years old with self-reported physical activity (PA) levels below national recommendations will be recruited from the Minneapolis-St. Paul, Minnesota metro region. Participants who respond to newspaper advertisements or fliers will call a central project telephone number to be scheduled for initial eligibility screening by the study coordinator.

Figure 3. Study Overview

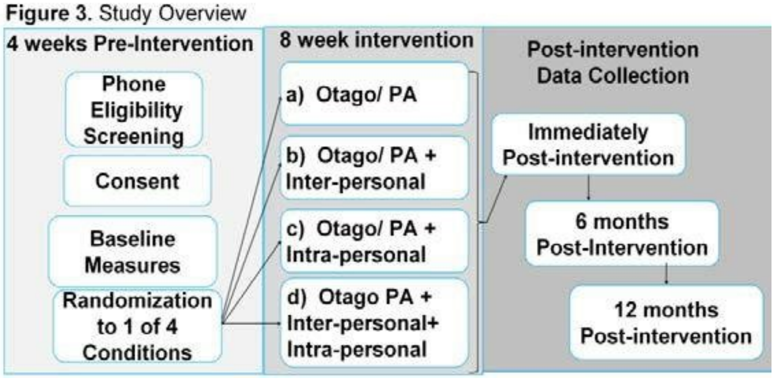

Screening. Potential participants who respond to newspaper advertisements or fliers will call a central project telephone number to be scheduled for initial eligibility screening by a trained study coordinator using the screening case report form in the Research Electronic Data Capture (REDCap). Screening questions will include:

- Age and date of birth
- History of lower extremity surgery
- Fall risk factors

- Three surveys:
    1. The Exercise and Screening for You (EASY)[56]
    2. The Rapid Assessment of Physical Activity (RAPA) [54]
    3. A six-item screener for cognitive function (name three items; recall three items; year; month; day of the week)

Consenting and Baseline Data Collection. If potential participants are eligible and interested, trained study staff will meet them in person to obtain written informed consent.

Once consenting is complete, the trained study staff will conduct baseline measures: (a) an interview including self-report measures (50 minutes); (b) observation of functional gait and balance (10 minutes); (c) initial or orientation to PA monitor--Fitbit (20 minutes); (d) after a one week practice period, participants and RA will meet again to confirm that the PA- monitor is acceptable and usable, and to begin the baseline 7 day PA data capture period (15 minutes); (e) after wearing the PA-monitor for 7-10 days, at least 10 hours each day, participants and study staff will meet again to complete the PA-data capture (15 minutes). Combined, the baseline procedures will require approximately 2 hours, and be completed over the course of 2 weeks.

Randomization: Once potential participants have provided consent and baseline data collection is complete, they will be formally randomized to 1 of 4 study conditions. They will begin the 8-week intervention within 8 weeks of enrollment/ randomization.

There will be rare situations when a participant is enrolled and randomized in the study, but is unable to begin or complete the 8-week intervention due to unforeseen changes in their life such as the death of a family member or the sudden need for medical treatments. In such situations, participants will be given the option to: (a) delay their participation in the 8-week intervention portion of the study, (b) withdraw from the 8-week intervention, but undergo one or more of the post-intervention data collection meetings, or (c) withdraw from all aspects of the study. Trained study staff will identify and track such situations in REDCap.

If participants choose option (a), they will **not** be re-randomized. Rather, researchers will invite them to an 8-week intervention course/ condition to which they were originally randomized, but that is scheduled to occur in a later time-frame.

Research staff will repeat screening to determine continued eligibility.

- If participants remain eligible, research staff will repeat baseline assessments within 8 weeks of the participant's delayed start of the 8-week intervention.
- If participants become ineligible---, reason(s) for ineligibility will be documented.

The analyst will compare and report the results of repeated baseline measures for subjects who choose option (a) and remain eligible for the study. If the differences between the repeated baseline measures are not significant, the first set of values will be used in inferential analyses. If the differences between the repeated baseline measures are significant, the second set of values (those most proximal to the intervention delivery) will be used in inferential analysis. A description of this will be included in all reports.

Research Outcome Data Collection. Trained research assistants will collect all data. They will be trained to accurately collect and manage data and to interview older adults in ways that are non-threatening, friendly, and respectful. Data will be derived from in-person interviews, observations, monthly fall calendars, and objectively measured PA parameters from accelerometers within participant's commercially available PA-monitors.

In-person interviews include scripted, structured interviews asking questions from these validated tools (all widely used) at the following time points:

- Baseline only: Demographic*
    - Biological
- Baseline; immediately post-intervention; 6 months' post-intervention; and 12 months post-intervention:
    - Pain via the Brief Pain Inventory-short form (Tan, 2004; Mendoza, 2006) [89,90]
    - Medication Inventory (see Appendix 2)
    - Self-reported health and Fall Risk (Stevens, 2013; Kunkel, 2011) [55,75]
    - Impact of the COVID-19 Pandemic and Accompanying Mitigation Efforts on Older Adults' physical activity and wellbeing[110]_
    - Social Support and Exercise Survey (Family and Friends) (Sallis et.al , 1987)[80]
    - Duke--UNC Functional Social Support Questionnaire*
    - Index of Readiness (Fleury, 1994) [22]
    - Self-Efficacy for Exercise (Resnick & Jenkins, 2000)[81]
    - Index of Self-Regulation (Fleury, 1998)[84]
    - Physical Activity Enjoyment Scale-8
    - Behavioral Regulation in Exercise Questionnaire (Markland & Tobin, 2004)[83]
    - Life-Space Mobility[111]
    - Physical Function-Short Form-6b (PROMIS; Rose, 2008)[79]
    - Physical Activity Scale For The Elderly PASE[72]
    - Health and Physical Activity Resources Survey (CIRS)[112]
    - Group Cohesion**
    - Quality of Life: Global Health Scale Patient-Reported Outcomes Measurement Information System, including items that address physical and social function (PROMIS) (Hays, 2009)[76]

*Administered at baseline only
**Administered immediately post-intervention only

Coronavirus adjustments: March 17, 2020
Assessment visits, which are structured interviews lasting approximately 45 to 90 minutes, will occur over the phone for the duration of new Coronavirus recommendations. We will notify the participants of this change. Our researchers will adjust their schedules according to our participants' availability and mail them information as needed.

The SPPB portion of the assessments, which require in person observation, will be delayed until Coronavirus restrictions are lifted.

Observations of functional strength and balance include the Short Physical Performance Battery at baseline; immediately post-intervention; 6 months' post-intervention; and 12 months post-intervention.[77] This battery includes timed repeated chair stand, semi-tandem, side by side, tandem balance testing, and a timed walk. Scores for each item are summed for a total score

ranging from 0-12 (12 indicates no impairments). The gradient of resulting scores validly reflects variation in lower extremity strength in older adult populations, and is predictive of progressive disability. It also demonstrates that the timed walk is predictive of catastrophic disability. The test will be discontinued if either the participant or the RA perceives it is unsafe to proceed.

Fall calendars will be sent to participants monthly with instructions to document injurious and non-injurious falls by day of the month, as well as related circumstances and consequences on the questionnaires, which are located on the back of each calendar.73,74 They are mailed to participants one week prior to the beginning of each month. Participants will document prospectively on the calendars throughout the month, and then return them to the research team in the stamped, addressed return-envelope provided. We will call participants who do not return calendars to identify and problem-solve barriers to using them. We will also call participants who report falls to further explore fall circumstances and consequences.

Objectively measured PA will be captured via the commercially available PA-monitors at baseline; immediately post-intervention; 6 months post-intervention; and 12 months post-intervention. In this study we will use Fitbit Ones™ unless they are not available; in which case we will choose another equivalent Fitbit model. Participants will be asked to wear their Fitbits during data collection periods for up to 10 hours daily, for 7-10 consecutive days. Each of their PA-monitors will be assigned an alphanumeric ID and will be registered under that ID, which is not connected to their study documents or personally identifiable information. Data, PA intensity classifications and number of steps walked (day total, hour, and minute-by-minute intervals) will be captured by Fitabase, a fully hosted, cloud-based software that implements robust industry standards to maintain secure research databases and keeps data private.[67] Fitabase enables the aggregation, reduction, visualization and management of data prior to downloading it into Research Electronic Data Capture (REDCap). Both databases meet privacy and security required when conducting research responsibly.

Process evaluation. Because this is a behavioral randomized control trial we will evaluate, formatively and summatively, processes involved with implementing the intervention as planned. In particular, we will (a) examine the extent to which the intervention was delivered as planned by each interventionist using indices of procedural consistency by the interventionist and by the principal investigator (PI) reviewing randomly selected audiotapes of intervention meetings, as well as field notes from each meeting; (b) the extent to which individual participants received and enacted the intervention using attendance records, tracking progress of each participant's' exercises (tracking tools for this are part of the evidence based PA protocol); and, (c) multilevel factors (e.g., individual, social, environmental and institutional) that may influence intervention implementation and effects using semi-structured interviews of randomly selected study participants (n=30), from the perspectives of leaders within community centers that host this intervention (n= 5). These interviews will be audiotaped and analyzed using content analyses.

Attendance after each meeting documented by the interventionist. If a participant misses more than one meeting, trained study staff will call them to discuss and problem solve any barriers to attendance.

The interventionists will document attendance and field notes after each meeting. They will also track individual participant progress with the exercises that are included in the evidence-based protocol, which is part of the standard administration procedures of the protocol.

After completing the 8-week intervention, participants will be scheduled for data collection meetings immediately post-intervention (e.g., during the week after the intervention ends); 6

months post-intervention; and 12 months post-intervention. Procedures used during these data collection meetings will be similar to the aforementioned baseline procedures, minus the 1 week practice period. Combined, post-intervention procedures will require 4.5 hours (90 minutes per data collection time point).

Semi-structure interviews to ascertain participants' perspectives of multi-level Barriers and Facilitators of implementation and effect immediately post study.

5.4 FOLLOW-UP

Please Refer to section 5.3 above

CHAPTER 6 – DATA BANKING

Not Applicable

CHAPTER 7 – SHARING OF RESULTS WITH PARTICIPANTS

7.1 DISSEMINATION

We will share protocol content and study findings with interested participants, community partners, and public health officials in Minnesota. Our dissemination plan has three main facets. First, we will publicly present information about all aspects of this proposal; study protocol; intervention content; recruitment and retention strategies; and findings local meetings within community centers. We will also make annual presentations locally through the UMN School of Nursing's Annual Research Conference; during meetings with community partners; and during Minnesota's annual Fall Awareness Day.[101] Second, we will develop a website for the study where we will post information about PA and falls and post summaries of our presentations on this Web site, written in plain language for a general audience. Third, we will provide participants with a report of their personal results pertaining to their balance, leg strength and PA.

CHAPTER 8 – STUDY DURATION

- The duration anticipated for an individual participant's participation in the study: **15 to 18 months**
- The duration anticipated to enroll all study participants: **4 years**
- The duration anticipated to complete all study procedures and data analysis: **6 years (given COVID-19)**

CHAPTER 9 – STUDY POPULATION

9.1 INCLUSION CRITERIA

All potential participants will be screened by trained study staff for inclusion based on the following criteria:

Inclusion Criteria:

- ≥70 years of age
- English speaking
- Low PA as determined by the Rapid Assessment of Physical Activity Survey[54]
    - Aerobic sub score 1-4 – Does not participate in endurance exercise such a walking more than 150 minutes per week, and / or
        - Strengthening/Balance activities sub score of 0 – Does not participate in strengthening and/or balance exercises more than once per week

- One or more fall risk factors as determined by CDC, Steadi fall risk screener [55]
    - One or more falls in the last year
    - Unsteadiness when standing or walking
    - Worries about falling
- Participants who self-report the following symptoms will require clearance from a primary provider (as guided by the Exercise and Screening for You Questionnaire):[56]
    - Pain, tightness or pressure in chest during PA (walking, climbing stairs, household chores, similar activities) *that have not been checked and/ or treated by a healthcare provider*
    - Current dizziness *that have not been checked and/ or treated by a healthcare provider*
    - Current, frequent falls *that have not been checked and/ or treated by a healthcare provider*

9.2 EXCLUSION CRITERIA

Exclusion criteria for participation in this study include:

- Lower extremity surgery within the past 6 weeks
- Inability to walk
- Formal diagnosis of neurocognitive impairment or Callahan Dementia Screener score of < 5.

9.3 SCREENING

Screening: A trained study staff will screen potential participants via telephone interviews using the screening case report form in Research Electronic Data Capture (REDCap), which includes questions about:

- Age

- History of lower extremity surgery Fall risk factors
- Three short surveys:
    1. The Exercise and Screening for You (EASY) [56]
    2. The Rapid Assessment of Physical Activity [54]
    3. The Callahan Screener for Cognitive Dysfunction [113]
        - Repeat the following words (apple, table car)
        - What is the year
        - What is the month
        - What is the day
        - Remember the words we just talked about (apple, table, car)

CHAPTER 10 – VULNERABLE POPULATIONS

This study does not include vulnerable populations; however, we will not exclude older adults with variable levels of functional capacity or the economically disadvantaged. Approximately 36% of older Americans report having difficulty performing one or more activities of daily living; and 9.5% report incomes below the poverty level. [108] Thus, these populations should not be excluded from intervention studies designed to increase PA, reduce falls, and improve QOL.

CHAPTER 11 – NUMBER OF PARTICIPANTS

11.1: NUMBER OF PARTICIPANTS TO BE CONSENTED

308

CHAPTER 12 – RECRUITMENT METHODS

12.1 RECRUITMENT PROCESS

.Recruitment strategies will combine ones employed in our successful preliminary study with strategies that can reach senior groups within the many neighborhoods from which we recruit. In collaboration with community partners and experts from the University of Minnesota (UMN) Clinical Translation Science Institute (CTSI), we will design advertisements and fliers to place in neighborhood and city-wide newspapers and locations frequented by older adults (e.g., coffee shops; clinics). Thus, recruitment efforts will extend to the immediate neighborhoods of the community centers as well as the surrounding townships and suburbs.

We will also create a study website and a Facebook page that will include information about the study for potential volunteers. The Facebook page content will be taken primarily from the current website. Through Facebook, we will reach out to senior groups who also have Facebook pages to let them know about the study.

If recruitment is slower than anticipated, we will employ an additional strategy for recruitment – we will send letter to mailings from Fairview (FV) to potential participants (see letter in recruitment attachments). This mailing process begins with a request to University of Minnesota's Clinical Data Repository for data to support preparatory research work (recruitment). Data from this request will include US mail addresses of individuals who are alive, over 70, and without dementia. Data will be de-identified to the PI and research team. The PI /research team will not contact people directly. Rather, Fairview/ UMN research services will be employed to send mailings to potential volunteers living in zip codes near community center hosting the study. Mailings will include a letter from FV (explaining the nature of the mailing, FV's role) and the letter from PI (briefly explaining the current study and how to learn more about it).

12.2: SOURCE OF PARTICIPANTS

Participants will be recruited from the community at large.

12.3: IDENTIFICATION OF POTENTIAL PARTICIPANTS

Participants will self-identify in response to the recruitment methods described in section 12.1 above.

Trained study staff, including study coordinator, RAs, and a licensed nurse, will make initial contact with potential participants.

No private or protected medical records will be used in this research.

12.4: RECRUITMENT MATERIALS

Refer to section 12.1 above and attached recruitment materials

12.5: PAYMENT

We will offer to coordinate and subsidize transportation to data collection and intervention meetings, upon request from participants in need.

Participants will receive $50.00 after each of the four PA data collection periods (approximately 7 days each) and $20.00 after each of the 4 data collection meetings (interviews).

The 8-week intervention meetings will be free of charge and participants will be encouraged to keep and use workbooks, ankle weights, and PA-monitors they receive as part of this intervention.

Forty participants will also be invited to participate in a semi-structured interview within 6 months of completing the study and will receive an additional $20.00 after that interview.

Therefore, the total possible compensation for participating in the study ranges from $280.00 to $300.00.

Compensation will go directly to the participant and Research Experience Points will not be awarded.

<u>Compensation schedule</u>. Participants will be reimbursed immediately after each data collection period as scheduled bellowed:

- Structured Interview 1 (Baseline): $20.00
- Structured Interview 2 (Immediately post-intervention): $20.00
- Structured Interview 3 (6 months post-intervention): $20.00
- Structured Interview 4 (12 months post-intervention): $20.00
- PA data collection period 1: (Baseline): $50.00
- PA data collection period 2: (Immediately post-intervention): $50.00
- PA data collection period 3: (6 months post-intervention): $50.00
- PA data collection period 4: (12 months post-intervention): $50.00
- <u>PROCESS Evaluation</u>: Post-study completion: $20.00
  - 25 to 30 participants (from each condition) will be selected, at random, to participate in a 30-45-minute interview addresses barriers and facilitators of intervention implementation and effect.

CHAPTER 13 – WITHDRAWAL OF PARTICIPANTS

13.1: WITHDRAWAL CIRCUMSTANCES

We anticipate all withdrawals from our study will be voluntary, based on our two pilot studies during which there were no involuntary withdrawals (out of 130 participants).

13.2: WITHDRAWAL PROCEDURES

When participants withdraw from the intervention, but continue to participate in data collection, we will:

- Document their withdrawal from the intervention and reason(s) for withdrawal (e.g., change in schedule, illness, family responsibilities)
  - We will use intention to treat, such that data from these participants will be included in the analysis.

13.3: TERMINATION PROCEDURES

Because the risks involved in this study are very low, we do not anticipate study termination. Should the study be terminated for administrative reasons, the study team will communicate to all participants, community partners and all study team members the timing and rationale for termination. Additionally, they will ensure data collected to date is managed, cleaned and available to investigators, IRB, NIH/NINR for reporting purposes.

CHAPTER 14 – RISKS TO PARTICIPANTS

14.1: FORESEEABLE RISKS

The minimal risks in this study relate to *psychological or social discomfort* as well as *mild temporary muscle soreness or discomfor*t.

The potential risk for minimal *psychological or social discomfort* may occur when completing surveys and attending small group intervention meetings. This risk is

considered minimal; the alternative is to not answer the questions or to not participate in the small group conversations that elicit discomfort.

The small potential risk for *mild, temporary muscle soreness or discomfort* when first performing the exercises. This is an expected, transient symptom and considered a minimal risk, not a serious risk. The risk of *injury* when performing exercises and movements in the Otago physical activity protocol is similar to risk of injury with everyday movements and activities of daily living, and considered to be a minimal risk. The alternative is to not engage in the exercises and movements in the Otago protocol or similar, which creates a risk of musculoskeletal disuse and, in turn, higher risks of falls.

Minimizing risk

To minimize research-associated risk, the protocol will be conducted fully in keeping with the manual of operation, approved by the IRB, and summarized in the consent form. The consent process informs each volunteer about the study, indicates the participation is voluntary and she/ he has the right to stop at any time. Risks are enumerated in the informed consent form and described verbally during the consent process. In addition, the design of the intervention integrates strategies to minimize risks:

> To minimize the risk of *psychological or social discomfort* we will implement three main strategies. First, all research staff successfully complete required training and maintain competence to facilitate 1:1 interviews (e.g., for data collection) and group discussions (e.g., intervention meetings) with older adults in ways that are non-threatening, friendly and respectful. Second, study participants will be reminded, verbally and via the written materials, that they do not have to complete any question they do not want to answer; that 1:1 interviews may be terminated at any time per their wishes; that they do not have to participate in any small group conversation they do not want to; and, that they are free to leave any intervention meeting at any time per their wishes. Finally, we will also inform all participants that they may choose to withdraw from the study at any time without negative consequences or without risking loss of present or future care they receive or access they have to community center resources.
>
> To minimize the risk of *mild, temporary muscle soreness or discomfort or injury* while performing exercises and movements in the study, we will implement three main strategies. First, the evidence-based physical activity protocol used in this study is effective and safe across older adult populations, including those with multiple chronic conditions and frailty. Second, participants will be informed, verbally (during intervention meetings) and in writing (via their workbooks) about what to expect, how to minimize risks of *mild, temporary muscle soreness or discomfort*, and how to prevent *injury.* Third, the PI has extensive experience leading Otago and is a board certified gerontological nurse practitioner. She and Becky Olson-Kellogg DPT, an expert in gerontological PT, will review training plan and monitoring for interventionists in this study. She will be available to assessors (e.g., for the SPPB) and interventionists for consultation throughout the study.

14.2: REPRODUCTION RISKS

Not Applicable

14.3: RISKS TO OTHERS

Not Applicable

CHAPTER 15 – INCOMPLETE DISCLOSURE OR DECEPTION

Not Applicable

CHAPTER 16 – POTENTIAL BENEFITS TO PARTICIPANTS

16.1: POTENTIAL BENEFITS

We believe participation in this research will yield benefits for participants. Mastery of physical activities within the Otago PA protocol will provide participants with skills and knowledge to practice these exercises safely at home or in other environments of their choice. The behavioral change strategies in the intervention have the potential to motivate participants continued engagement in PA. Participants will also receive a PA-monitor for self-tracking and support to use it as well as interpret its data.

CHAPTER 17 – DATA MANAGEMENT

17.1: DATA ANALYSIS PLAN

Statistical analysis. Descriptive statistics (means, standard deviations, ranges for continuous variables and counts/ percentages for categorical variables) will be used to summarize biologic, demographic, outcome, and exploratory measures by treatment arm, actual and projected accrual, attendance and retention rates, quality control data (e.g., missing data), and fidelity data. Tables, graphs and charts will be used to visualize data when appropriate. Enrollment summaries will be reviewed at weekly research staff meetings throughout enrollment phases and every month during non-enrollment phases. Differences by randomly assigned condition will be assessed with one-way ANOVA tests for continuous and Fisher's exact test or chi-square tests for categorical variables as appropriate. Any identified confounders will be adjusted for in subsequent analyses. All analyses will be pre-planned and conducted while masked to condition.

We will include all cases in analyses, regardless of intervention attendance rates or attrition. Missing data will be analyzed. We anticipate, based on preliminary research that the rate of missing data will be low and similar across conditions, but that it may be associated with severe illness that leads to dropout or inability to capture follow- up PA data. Thus, similar to preliminary analyses, we will conduct a sensitivity analysis of complete cases and imputing worst case carried forward.

Primary Aim: Intervention component effects on PA will be assessed using 2x2 full factorial ANCOVA models with duration of total PA as the outcome (average minutes per

week). The factors, or independent variables, are interpersonal and intrapersonal components; each with two levels indicating exposure (No vs Yes). Different models will be developed for PA measured objectively. PA will also be measured via self-report. Each model will include baseline PA values as a covariate. Main and interaction effects of the components on the duration of PA will be tested immediately, 6 months, and 12 months post- intervention. Further, we will apply multilevel longitudinal data analyses to examine the variation of component effects across time-points (baseline; immediately post-intervention; 6 months' post-intervention; and 12 months' post- intervention), including interactions between components (e.g. interpersonal x intrapersonal x time), which will allow us to see when an older adult might benefit from additional support to maintain PA.

Secondary Aim: The secondary outcome of Falls will first be described using the number of falls, fallers, fall rate, and time to first fall post-intervenion.[74] Given the expected distribution of falls, we will use negative binomial regression models to estimate the between-group difference in fall rates 12 months' post-intervention. Quality of Life data will be assessed using 2x2 full factorial ANCOVA and longitudinal analyses described for Aim 1 with total mean QOL as the outcome variable.

Exploratory Aim: The exploratory aim of the study is to investigate the nature of relationships between receipt of one or more intervention component and outcome variables. We will assess if this study's intervention component(s) changed mediator variables, and in turn caused increased PA or reduced falls. In this study, the putative mediators of PA are Motivational Constructs: a) social support (support from family and friends for physical activity); b) readiness (Index of Readiness, Self-Efficacy for Exercise Scale); and c) self-regulation (index of self-regulation, goal attainment, Behavioral Regulations in Exercise Questionnaire-3). The putative mediators of falls are Physical Markers of Fall Risk: leg strength and balance (SPPB, Physical Function SF 6b). Using the general approach to mediation analysis described by MacKinnon,[96] we will assess the effects of intervention components on mediators, and the effects of mediators on outcomes, as well as the total direct and indirect effects of intervention components on PA and falls. Counterfactual approaches described by Vanderweele and colleagues[97] will be used if nonlinearities or interactions between exposure and mediator variables are observed.

Additional exploratory analyses: pain, medication, and sex as a biological variable: Although this study is not designed to conduct confirmatory subgroup analyses of covid-19, pain, medication, or sex,[98,99] we will conduct stratified, exploratory analyses to assess effect modification by these variables. We will report values for these variables as well as differences observed, which may be valuable for future research and meta-analyses

17.2: POWER ANALYSIS

Sample size and power. The primary aim of the proposed study is to detect clinically meaningful intervention effects on PA from baseline to 12 months' post-intervention. Meta- analyses in the field of PA and older adults describe standardized effect sizes that range from .18 [31] to .26[, 92] which translate to 670 to 870 additional steps per day or 73 to 94 additional minutes of PA per week. Although these effects are small, they are considered to be clinically important in older populations whose overall activity levels are low; often sedentary.[93] Thus, we based our sample size estimates on a small

standardized effect size of .20. We also anticipate 15% attrition at 12 months, based on our preliminary studies which showed 7% attrition at 6 months. Considering these assumptions, a sample size of 308 (77 per condition) will enable us to detect small but clinically meaningful main effects of the interpersonal or intrapersonal components as well as their interaction (effect sizes of .20), with 80% power, under a two-tailed hypothesis test at a significance level of .05.[94] This sample size will also enable us to detect medium to large effects on our secondary outcome of falls (fall rate ratios of .40 to .70), with 80% power, under a two-tailed hypothesis test, at a significance level of.05, which is consistent with prior research that shows Otago PA reduces falls (fall rate ratios 0.56 to 0.79).[10]

17.3: DATA INTEGRITY

Data Monitoring. Ongoing quality control procedures will be implemented for data collection, storage and processing. The data manager will conduct monthly monitoring of the study database and generate a report for the PI to review at team meetings. Standing agenda items for these meetings will include participant recruitment and retention, serious adverse events, unexpected adverse events, expected adverse events, protocol deviations, data integrity, and overall study conduct.

CHAPTER 18 – CONFIDENTIALITY

17.4: DATA SECURITY

Data Collection. All data from participants screened for the study will be entered and stored in an electronic study database. Designated research staff will collect and enter required data (written informed consent, responses to interview data) onto electronic study data forms. Screened participants who do not meet study eligibility will have specific screening data entered the study database as this data will be helpful in examining the patient population and feasibility of enrollment criteria and will include gender, age, race and reason for exclusion. All dates will be shifted and other Personal Health Information (PHI)--which in this study includes self-reported medical history, fall risk, and medication regimen--will be removed from the study database upon study completion. All data obtained from this study will be used for research purposes only and will not include data from electronic health records.

Interview and observational data will be entered directly into a Research Electronic Data Capture (REDCap) database from encrypted, password protected iPad tablet computers. Physical activity data will be downloaded into REDCap (link to REDCap at University of Minnesota: https://sites.google.com/a/umn.edu/redcap-help/) from Fitabase (link to Fitabase: https://www.fitabase.com/), a research database for accelerometer data captured from Fitbit. Fitabase captures, aggregates, and securely stores de-identified accelerometer data from Fitbit monitors. Participants receive unique Fitbit research identification numbers; different from their study identification numbers and personal identifying information. Our team has extensive experience using both REDCap and Fitabase in previous research.

Case Report Forms. All proposed study specific case report forms (source documents) for data collection will be designed by the PI within the electronic Case Report Forms (eCRFs) for use in the study's REDCap database. All study specific eCRFs source documents and collected data will be organized by the participant's unique study

identification number in the participant research record, available only to the PI and study coordinator. Completed paper consent forms that require signature will be scanned and uploaded into the study database as well as maintained on file in accordance with University of Minnesota Institutional Review Board (IRB) policies and applicable National Institutes of Health (NIH) Federal Regulations for the Conduct of Human Participant Research.

Binders. The study coordinator will prepare and maintain a binder for each participant containing all non- eCRFs records. Regulatory files will also be maintained to include the IRB- approved Protocol, original Informed Consent documents, and other study-related regulatory documents.

Binders and all study related records will be stored in a locked file cabinet within a School of Nursing research office located on the University of Minnesota campus. The research office and the building housing this office, (Dinnaken, room 120, 925 Delaware St SE, MPLS, MN 55414), are both secured 24 hours per day, 7 days per week. Also, the building and the nursing research office within the building are access controlled with card readers, granted by the PI only. Additionally, the building is monitored by the University of Minnesota security services 24 hours per day, 7 days per week. This building also houses research offices for other departments and centers in the University of Minnesota such as the Transplant Research Organization.

Access to the research records, study database and PHI's will be restricted to study personnel as approved by the PI and IRB. As with all studies conducted at the University of Minnesota, this study is also eligible for a random audit by University of Minnesota's Office of Compliance, as well as NIH, National Institute of Nursing Research (NINR).

Data Processing. This study will use REDCap for data capture and management.

Data Security. Data entered directly into our REDCap database via tablet computers (e.g., IPad) used exclusively for research, which are encrypted and protected by strong passwords. We will use a University of Minnesota, School of Nursing established policy and procedure for using research-only computer tablets that includes details about security, access to internet, and access to REDCap, transport, and storage. When not being used in data collection meetings, these research-only tablet computers will be stored in research office space housed in the Dinnaken Office Building. Details about the security and monitoring of this office and building are described above under Binders.

REDCap uses a MySQL database via a secure web interface with data checks used during data entry to ensure data quality. REDCap includes a complete suite of features to support HIPAA compliance, including a full audit trail, user-based privileges, and integration with the institutional Lightweight Directory Access Protocol server. The MySQL database and the web server will both be housed on secure servers operated by the University of Minnesota Academic Health Center's Information Systems group. The servers are in a physically secure location on the University of Minnesota campus and are backed up nightly, with the backups stored in accordance with the AHC-IS retention schedule of daily, weekly, monthly tapes retained for 1 month, 3 months, and 6 months, respectively. Weekly backup tapes are stored offsite in Minnesota. The AHC-IS servers provide a stable, secure, well-maintained, and high-capacity data storage environment, and both REDCap and MySQL are widely-used, powerful, reliable, well-supported systems.

Data Entry. Each participant will be assigned a unique study identifier in REDCap, all PHIs will be masked, and data exports will be limited to the PI and the data manager for generating reports and the conduct of statistical data analysis.

Security and privacy information: a statement from Fitabase. As a research platform that collects data from internet connected consumer devices, Fitabase takes security and privacy seriously. Fitabase is a fully hosted, cloud-based software solution that implements robust industry standards to maintain secure databases and keep data private. Fitabase code and databases physically reside on the Microsoft Windows Azure platform (www.windowsazure.com). We rely on the robust security, both physical on premise guarding, and over network, provided as part of that platform. From Microsoft (http://www.windowsazure.com/en-us/support/trust-center/security/): Windows Azure runs in data centers managed and operated by Microsoft Global Foundation Services (GFS). These geographically dispersed data centers comply with key industry standards, such as ISO/IEC 27001:2005, for security and reliability. They are managed, monitored, and administered by Microsoft operations staff that have years of experience in delivering the world's largest online services with 24 x 7 continuity. In addition to data center, network, and personnel security practices, Windows Azure incorporates security practices at the application and platform layers to enhance security for application developers and service administrators. In addition to the primary copies of our databases, Small Steps Labs LLC maintains snapshot archives of our database for disaster recovery purposes. Our backup copies reside on hardware only accessible to Small Steps Labs LLC and our employees. Our backup copies are encrypted and password protected. Fitabase uses Secure Sockets Layer (SSL) for all authentication (logins), billing, and administration of the site. The user's browser establishes the authenticity by requesting an SSL certificate that verifies the identity of Fitabase. Once that SSL certificate is recognized, a Secure Sockets Layer (SSL) connection is established for security, encrypting data transmitted between browser and web server. Passwords: Fitabase stores passwords in encrypted form. When an administrator attempts to log in to Fitabase.com, their attempted password is encrypted and if matched, the user is allowed in to the site. This practice prevents unauthorized usage of the site. If the database were to be compromised, passwords would not be retrievable. Fitabase logs all site usage, including attempts to access restricted data, or log in to accounts of others. We maintain security policies to block / freeze accounts that appear to be compromised until we are able to make contact via the email address used to set up the administrator account. Fitabase allows groups wishing to collect data anonymously the option to do so by associating device data with their own alphanumeric identifiers. To best accomplish this, groups should set up the Fitbit.com account that corresponds with each device using an anonymous email address not linked to a real person. Fitabase does not collect personally identifiable information beyond what it is provided by Fitbit.com.

Additionally Fitabase does not collect IP addresses from synced participant devices. Fitabase stores information provided to it by the Fitbit API (dev.fitbit.com). This information about:

Data is stored and indexed in the Fitabase SQL Server database in day total, hour, and minute-by-minute intervals. Our database servers are IP firewalled and whitelisted such that they refuse any connection from IP addresses not preprogrammed by us. No GPS or other location information is collected.

CHAPTER 18 – PROVISIONS TO MONITOR THE DATA TO ENSURE THE SAFETY OF PARTICIPANTS

This study introduces a minimal risk to participants,

18.1: DATA INTEGRITY MONITORING

The Data Safety Monitoring Plan (DSMP) for this trial will focus on close monitoring by the PI in conjunction with research staff and an Independent Safety Monitor who will be available to review and recommend appropriate action regarding individual reports of serious adverse events, adverse events, and other safety issues.

The Principal Investigator (PI). The PI will have the primary responsibility to monitor this study. The PI will review data on such aspects as participant enrollment, study procedures, forms completion, data quality, losses to follow-up, and other measures of adherence to the protocol. The PI will review all possible and real events, and seek recommendations from the Independent Safety Monitor in real time regarding all serious adverse events and adverse events. The PI will also be responsible for submitting necessary reports to NINR and the University of Minnesota institutional IRB within 72 hours and 5 working days of each serious adverse event and adverse event.

Research Staff. The trained and IRB certified research staff, including RAs, a study coordinator and interventionist, will continuously monitor safety and immediately apprise the PI of all possible or real events so that appropriate communication and action can be taken in a timely manner. The data manager will prepare tables with data related to enrollment, retention, adherence on a monthly basis for review by the PI and her research team.

Independent Safety Monitor: Diane Treat Jacobsen PhD, FAAN, Chair of the University of Minnesota's School of Nursing Adult and Gerontological Cooperative. She is an adult / gerontological clinical nurse specialist and experienced researcher in randomized, controlled intervention studies for older adults that focus on physical activity to reduce peripheral arterial disease, as well as quality of life. She has received external funding for more than 8 years to support her research in clinical and community settings. Dr. Treat-Jacobsen is independent of the current study and is available in real time to review and recommend appropriate action regarding serious adverse events, adverse events, and other safety issues.

The primary responsibilities of the safety monitor will be to evaluate the performance of this study, the safety of the participants, and to determine if continuation of the study is appropriate; scientifically and ethically. The PI will have several meetings with the safety monitor. The first meeting will be held prior to recruiting participants to evaluate the protocol, consent process and analysis plan. The second meeting will occur after 30 participants have enrolled (10% of expected enrollment) and then every six months to review progress on this project. The PI, and the safety monitor will also meet on an as-needed basis to review any possible or real adverse events.

18.2: DATA SAFETY MONITORING

The investigators of this study have established a data safety and monitoring plan. Data Monitoring. Ongoing quality control procedures will be implemented for data collection, storage and processing. The trained study staff will conduct monthly monitoring of the study database and generate a report for the PI to review at team meetings. Standing agenda items for these

meetings will include participant recruitment and retention, serious adverse events, unexpected adverse events, expected adverse events, protocol deviations, data integrity, and overall study conduct.

Monitoring Study Safety. To monitor safety, indicators for each aspect of the scientific process will be tracked in real time then summarized and evaluated on a monthly basis. Aspects of the scientific process that will be tracked include initial screening of participant by inclusion and exclusion criteria; adherence to informed consent procedures; provision of study instructions to participants; provision of instructions to research staff for Good Clinical Practices, regulations pertaining to the Conduct of Human Participant Research, intervention delivery, and weekly as as-needed contact with participants for troubleshooting technology; protocol fidelity monitoring; and all possible serious adverse events and adverse events.

Data Monitoring. Ongoing quality control procedures will be implemented for data collection, storage and processing within REDCap. The trained study staff will conduct monthly monitoring of the study database and the data manager will generate a report for the PI to review at team meetings. Standing agenda items for these meetings will include

- participant recruitment and retention
- serious adverse events
- unexpected adverse events
- expected adverse events
- protocol deviations
    - Screening
    - Enrollment
    - Randomization
    - Intervention delivery
    - Data collection
    - Privacy protection
    - Confidentiality
- data integrity
- overall study conduct

Monitoring schedule. Safety data will be collected real-time, and summarized and reviewed monthly by the PI, research team and investigator teams.

Auditing selected cases. The research team will be prepared to accommodate requests from NINR or University of Minnesota IRB, as well as NIH, NINR to audit selected cases for compliance with IRB requirements, conformance with informed consent requirements, verification of source documents, and investigator compliance.

Adverse Events. Potential risks identified for participants are outlined in the Protection of Human Participants and will also be outlined in the IRB-approved informed consent document. Additional unknown risks may occur and, if so, will be identified through weekly diligent monitoring by the PI throughout the conduct of this study. During the informed consent process, participants will be advised of the potential minimum risks of participation as identified in the IRB-approved informed consent document. Also, participants will be reminded to promptly inform the researchers about any concerns regarding potential serious adverse events or

adverse events during intervention meetings, during data collection meetings, and via written materials: participant workbooks, meeting schedules, fall calendars. Participants will also be instructed to notify the PI and/or designee of any *suspected* serious adverse events or adverse events immediately, if possible. The PI will maintain an electronic record of all reported serious adverse events and adverse events. In addition to recording events, the PI will notify the Independent Safety Monitor of all reportable events as they occur, as well as submit this information to the UMN IRB and NIH, NINR.

Procedures for reviewing adverse events and unanticipated problems. Serious adverse events, expected adverse events, and unexpected adverse events will be assessed and graded by Independent Safety Monitor using the University of Minnesota IRB's Adverse Event Reporting Policy (http://www.research.umn.edu/irb/guidance/ae.html) and these criteria and this information will be given to the UMN IRB, as well as NIH, NINR within 5 working days of the event:

- Serious—an adverse event that results in death, is life threatening, or places the participant at immediate risk of death from the event as it occurred, requires or prolongs hospitalization, causes persistent or significant disability or incapacity, or Is another condition which investigators judge to represent significant hazards.
- Expected/Anticipated—Identified in nature, severity, or frequency in the current protocol, informed consent, investigator brochure, or with other current risk information.
- Unexpected/Unanticipated—not identified by nature, severity or frequency in the investigator's brochure, or current University IRB-approved research protocol or informed consent document, taking into account the characteristics of the participant population being studied.

We anticipate, based on health U.S. health trends and consistent with our preliminary research, that 45% the study population will have more than two chronic conditions, 25-76% will have pain, 85% will take more than one medication while 35% will take more than 5 medications. Thus, we will focus on determining the relationship of serious adverse events, expected adverse events, and unexpected adverse events to behavioral intervention being tested, categorized as:

- Unrelated—there is not a reasonable possibility that the adverse event may have been caused by the behavioral intervention being tested.
- Possibly related—the adverse event may have been caused by the behavioral intervention being tested, however there is insufficient information to determine the likelihood of this possibility.
- Related—there is a reasonable possibility that the adverse event may have been caused by the behavioral intervention being tested.

Procedures for identifying adverse events and unanticipated problems. The PI will be responsible for ensuring that all serious adverse events, expected and unexpected adverse events are reported to the University of Minnesota's IRB, as well as NIH, NINR in compliance with their requirements. Within 72 hours after a reportable serious adverse event, expected or unexpected adverse event has been reported by the participant or study staff, it will be graded by the PI, forwarded to the study's Independent Safety Monitor for review, and submitted by the PI to the University of Minnesota's IRB. The Institutional Official(s) will review the event and discuss the report with the IRB Chair and the Director of the Office of Research Integrity. Within 72 hours, after IRB review and acknowledgement, the PI will forward a copy of the reportable

serious adverse event, expected adverse event and unexpected adverse event, together with the IRB acknowledgement letter to the NIH, NINR Program Officer through the University of Minnesota's School of Nursing, Office of Research. In addition, all cumulative reportable serious adverse event, expected adverse event and unexpected adverse event will be included in the PI and research team's monthly reports and will be submitted to the University of Minnesota's IRB and NIH, NINR in the PI's Annual Progress Reports.

Examples of Potential Reportable Adverse Events. Serious adverse event, expected adverse event, and unexpected adverse events are reportable if they meet all of the following criteria: (a) is serious and/or suggests that the research places participants or others at a greater risk of psychological or physical harm than was previously known or recognized (b) is related and/or possibly related, or (c) is unexpected. Additionally, per University of Minnesota's IRB policy, all participant deaths, protocol deviations, complaints about the research, and breaches of confidentiality are reportable events.

An example of an adverse event would be a breach of physical activity data, which has never happened in any of our previous studies. We have taken numerous steps to prevent this. The physical activity data is online, so there is no paper file to access. Each participant has a Fitbit One™ registered using a unique research, not personal, name and password. Data; physical activity intensity classifications and number of steps walked (day total, hour, and minute-by-minute intervals) for each participant are captured by Fitabase, a fully hosted, cloud-based software that implements robust industry standards to maintain secure research databases and keeps encrypted data private (link to Fitabase: https://www.fitabase.com/). Fitabase enables the creation of unique Fitbit identification numbers, as well as aggregation, reduction, visualization and management of data--prior to downloading it into Research Electronic Data Capture (REDCap) (link to REDCap at University of Minnesota: https://sites.google.com/a/umn.edu/redcap-help/). Both Fitabase and REDCap meet privacy and security required when conducting research responsibly. Were someone to gain access to the physical activity data, they would not be able to identify the participants because these files use unique Fitbit identification numbers only, not study identification numbers or personally identifying information. If this unlikely event happens, we will report it using procedures outlined above. Steps would then be taken to identify how the breach occurred and what needs to be done to correct it.

An example of a serious adverse event includes the possibility of physical or psychological distress during the administration of the Short Physical Performance Battery (SPPB), an observational measure of functional strength and balance. To minimize this risk, research staff are trained and their continued competence to perform the SPPB, according to a standard protocol, is monitored monthly. The standardized protocol incudes instructions to elicit participant's comfort and willingness to proceed with each movement. If the participant or the research assistant are not comfortable proceeding, the movement is not pursued. Participants are offered a verbal and written copy of their SPPB scores. The SPPB is established as a safe test to use across older populations. Participants in our studies have not had serious adverse events, expected adverse events or unexpected adverse events during the SPPB. If this unlikely event happens, we will report this using procedures outlined above. Steps would also be taken to identify how the event occurred and what needs to be done to correct it.

Another example of a serious adverse event would be the death of a participant from chronic heart failure. While viewed as unexpected and unrelated to the behavioral intervention, death

from heart failure would nonetheless be a reportable, serious event. No further steps would be taken except to review, grade and report the event using procedures outlined above.

An example of an unanticipated problem would be if a participant became immobile during their participation in the study. As immobility is an exclusion, we would withdraw the subject from the study. This event will be reviewed, graded, and reported using procedures outlined.

Assessment of External Factors

The PI will conduct a semiannual assessment of external factors through a review of literature related to new developments in the areas of fall prevention, fall-reducing physical activity, behavior change strategies, and other approaches that may have an impact on the safety of participants or on the ethics of the study.

Interim Analysis

This study aims to test the effects of behavior change strategies combined with an evidence-based physical activity protocol. As such, the PI and study coordinator will generate semi-annual qualitative interim analysis reports on data obtained during phone calls and end-of-study surveys to understand issues related to the uptake, usability, and adoption of this intervention among this population: community-dwelling older adults. We will evaluate the screening and enrollment procedures, barriers to participation and retention, functional impact, acceptability, technology problems encountered if any, and user feedback from the participants, interventionists and community centers. Information gained from this structured process will be used to both guide the refinement of the current protocol and to inform the design of a larger implementation trial. There are no planned stopping rules for this study.

CHAPTER 19 – PROVISIONS TO PROTECT THE PRIVACY INTERESTS OF PARTICIPANTS

19.1: PROTECTING PRIVACY

Several facets of our research plan contribute to the protection of participants' privacy interest. First, trained research staff will interact with study participants. Only staff responsible for scheduling intervention meetings or data collection meetings will have formal access, via REDCap, to personal information such as address and phone numbers

Study Staff Training

Curricula and enduring training materials will center on a) the interventionist role; b) data collection and management; and c) PA-monitor support. Interventionists will be trained to deliver all manualized intervention content and gain these core competencies (renewed on an annual basis): facilitating small group discussions; maintaining a positive social milieu; delivering interpersonal (5) and intrapersonal behavioral change strategies (5); and delivering Otago.

Research assistants will be trained to collect and manage data and gain these core competencies: conduct structured interviews with older adults in ways that are non-threatening, friendly, and respectful and that enable the accurate collection of data for questionnaires and physical measurements; the accurate documentation and storage of data data using secure

iPads and REDCap. Additionally, RAs will also be trained to manage PA-monitors by gaining these core competencies: PA-monitor set-up; initial 1:1 orientation of participants to PA-monitor use; assist participants to troubleshoot PA-monitor; secure PA data collection and storage using the Fitabase research database.[67]

Experts within our research team will evaluate training curricula prior to delivery. The courses will be delivered via a combination of online and in-person strategies. All study staff will have continual access to the training course, manuals of procedures, and to the PI for reference and consultation as needed, as well as a Physical Therapist who specializes in older adult physical function, the SPPB, and the Otago protocol.

Finally, all study staff are trained regarding the responsible conduct of research, REDCap use, and good clinical practice for social and behavioral research.

To ensure good practices are maintained throughout the study during intervention meetings and data collection meetings, all study staff will be directly and indirectly supervised. Supervision will include observing that privacy of each participant is protected. Monthly process reports include a summary of privacy protection.

Second, intervention meetings will occur in rooms reserved for small group meetings. Such rooms are private, in that conversations within these rooms can only be heard by small meeting participants (study volunteers and trained interventionists). Visitors or other community center personnel or members will not be involved with these meetings. Interventionists have skills and abilities to facilitate small group conversations so that privacy is protected.

Third, data collection meetings will take placed between trained study staff, research assistants, and individual participants. These meetings will occur either in a private room within a community center or within the participant's home, whichever is preferred by the participant.

19.2: ACCESS TO PARTICIPANTS

This project does not require access to medical records, student records, or any other sources of private information. However, the project does entail collected self-reported health as well as names and addresses, which are needed to coordinate intervention delivery, fall calendar mailings and data collection meetings. Date of birth is needed to calculate age at the beginning of the study, which is a demographic baseline characteristic; the aggregate of which we will report as part of the study findings.

Not Applicable

CHAPTER 20 – CONSENT PROCESS

20.1: CONSENT PROCESS (When consent will be obtained):

Consent will occur between screening and enrollment. Within 10 days of being screened for eligibility, those potential volunteer participants who qualify for participation and remain interested, will receive a copy of the informed consent for the study via US mail

and meet in person with study staff who are trained in consent procedures. During the informed consent meeting, the trained will explain the project in detail to each potential participant. The study will be described, including its purpose; procedures, data collection, randomization, the intervention; use of the PA-monitor; potential risks and benefits; time required; and who to contact if there are concerns about the study. Eligible participants, their families, partners, or providers will be encouraged to ask questions or meet with the PI privately to further clarify questions, prior to providing consent. The participant may also take the consent form home again to look it over and ask questions of the trained research or PI at a time that is convenient to them. Interested persons will be under no time constraints to join or decline their participation.

Participants who provide verbal and written consent will be enrolled in the study. The consent form will also be signed by trained study staff or the PI, Dr. McMahon and filed in REDCap.

In addition to obtaining consent prior to study enrollment, ongoing consent will be ensured. During data collection meetings, trained research staff will remind participants that answering all questions is voluntary: they may refuse to answer any question within the interview and may discontinue the interview at any time. Similarly, the trained interventionist will remind participants during small group meetings that participation in intervention meetings is voluntary: they may choose to not participate or leave any of the 8 intervention meetings.

20.2 COMPENSATION OF RESEARCH RELATED INJURY

Not Applicable

20.3 CONTRACT LANGUAGE

Provide a copy of the contract language, if any, relevant to compensation for research-related injury.

20.4: WAIVER OR ALTERATION OF CONSENT PROCESS

Not Applicable

20.5: NON-ENGLISH SPEAKING PARTICIPANTS

Not Applicable

20.6: PARTICIPANTS WHO ARE NOT YET ADULTS

Not Applicable

20.7: COGNITIVELY IMPAIRED ADULTS, OR ADULTS WITH FLUCTUATING OR DIMINISHED CAPACITY TO CONSENT

Not Applicable

20.8: ADULTS UNABLE TO CONSENT

Not Applicable

CHAPTER 21 – SETTING

21.1: INTERNATIONAL RESEARCH

Not Applicable

21.2: COMMUNITY-BASED PARTICIPATORY RESEARCH

We will conduct the proposed study in community centers located in the four quadrants of Minneapolis (see letters of support from Tweed, Marinkov-Omorean, and Schoenberger within the supporting documents in ETHOS).

All research will be performed by UMN key personnel and staff, under the auspices of the UMN IRB. Community centers will host meetings during which UMN research staff will collect data and conduct intervention meetings. We chose these 4-community center locations for 4 primary reasons. First, we have longstanding partnerships (3+ years) with each center that include the conduct of our preliminary studies. Second, each community center has goals that are congruent with this proposal's objective. Third, each center serves ethnically and socioeconomically diverse populations. Fourth, each center has environments conducive to small group discussions and practicing fall-reducing PA, is accessible via public transportation, and has free parking.

Each center will host the small-group intervention meetings during 2 to 4 of the 13 study waves. Community-research teams will establish times and days for intervention meetings one month prior to the first intervention meeting. Although meetings will be hosted within the community centers, staff there will not coordinate participant attendance, collect data, lead, or participate in the small group meetings. The centers are listed below:

- Sabathani Community Center, Minneapolis, Minnesota
- Volunteers of America, Minneapolis, Minnesota
- Southwest Community Center, Minneapolis, Minnesota
- Park Elder Center, Minneapolis, Minnesota
- East Side Neighborhood Services, Minneapolis, Minnesota
- St. Paul Parks and Recreation locations including Highland Park Community Center; Linwood Community Recreation Center, Palace Community Center
- Minneapolis Public Libraries including Hosmer and Pierre Bottineau
- Lutheran Redeemer Church, Saint Paul, Minnesota
- West 7th Community Center, Saint Paul, Minnesota
- University of Minnesota Urban Research and Outreach-Engagement Center, Minneapolis, Minnesota

We chose these community center locations for 4 primary reasons. First, we have longstanding partnerships (3+ years) with each center that include the conduct of our preliminary studies. Second, each community center has goals that are congruent with

this proposal's objective. Third, each center serves ethnically and socioeconomically diverse populations. Fourth, each center has environments conducive to small group discussions and practicing fall-reducing PA; is accessible via public transportation; and has free parking.

The PI and community center leaders will meet prior to the beginning of the study and then quarterly and as-needed meetings throughout all phases of this project. Similar to our preliminary studies, the research – community teams will review recruitment and retention plans; establish communication procedures for the reservation of private rooms for data-collection and intervention meetings; and local dissemination based on each center's preferences. Each year, one of the quarterly meetings will include a project progress report.

Additionally, the research team will interview a representative from each community center about facilitators and barriers to providing this program in the future. Interview topics will be framed using the *reach- effectiveness –adoption- implementation-maintenance* framework (RE-AIM), a system for evaluating this intervention's potential for translation.

Finally, we will seek advice about our community engagement at least every six months throughout the study from Milton Eder, PhD and experts in the UMN, *CTSI's Community Engagement to Advance Research and Community Health* (see letter of support).

21.3: RESEARCH SITES

The research will be conducted and administered through the University of Minnesota, School of Nursing. Potential research participants will be identified and recruited from the communities within Minneapolis, primarily through newspaper advertisements.

Research procedures will be performed primarily in the University of Minnesota, Nursing Research Space.

- The School of Nursing is housed in Weaver-Densford Hall, which includes approximately 35,000 square feet of teaching, research, and office facilities. Space for Dr. McMahon and her research team is also available at the 120 Dinnaken Office Building located approximately four city blocks from faculty and administrative offices. Available resources within the School of Nursing include offices, conference rooms, telephone, WebEx, fax, and secure confidential data storage areas.
    - Dr. McMahon's office in the School of Nursing is approximately 125 square feet and is equipped with a personal computer (including the necessary statistical software), laser printer, Web camera, telephone access, and ample locked file space to conduct the proposed evaluation. The computer has LAN access
    - Dr. McMahon and trained study staff for this project also have 4 research offices at the 120 Dinnaken Building on campus, with work space, computers, telephone access, and secure file cabinets for

Additionally, private rooms will be used by trained research staff to conduct data collection interviews and deliver the small group intervention. Private rooms will be reserved for these meetings within the following community centers, per participant availability and intervention schedules. These community centers do not have site-specific regulations or customs affecting the research. Because each community center is not conducting the research, they do not require local scientific or ethical review:

CHAPTER 22 – MULTI-SITE RESEARCH

N/A

CHAPTER 23 – REFERENCES

1. Centers for Disease Control and Prevention. Reporting system (WISQARS). *National Center for Injury Prevention and Control, Centers for Disease Control and Prevention (producer).Available from: URL: www.cdc.gov/ncipc/wisqars*.

2. Campbell AJ, Borrie MJ, Spears Gf, Jackson SL, Brown JS, Fitzgerald JI. Circumstances and consequences of falls experienced by a community population 70 years and over during a prospective study. *Age Ageing*. 1990; 19(2):136-141.

3. Tinetti ME, Williams CS. Falls, injuries due to falls, and the risk of admission to a nursing home. *N Engl J Med*. 1997; 337(18):1279-1284.

4. Liddle J, Gilleard C. The emotional consequences of falls for older people and their families. *Clin Rehabil*. 1995;9(2):110-114.

5. Hartholt KA, van Beeck EF, Polinder S, et al. Societal consequences of falls in the older population: Injuries, healthcare costs, and long-term reduced quality of life. *The Journal of Trauma and Acute Care Surgery*. 2011;71(3):748-753.

6. Gillespie LD, Robertson MC, Gillespie WJ, et al. Interventions for preventing falls in older people living in the community. *The Cochrane Library*. 2012. *Cochrane Database Syst Rev* 2, no. CD007146. 2012

7. Centers for Disease Control and Prevention. Wide-ranging online data for epidemiologic research, DATA2010 the healthy people 2010 database; focus area 22-physical activity and fitness. http://www.healthindicators.gov/Indicators/Selection.

8. Hughes SL, Leith KH, Marquez DX, et al. Physical activity and older adults: Expert consensus for a new research agenda. *Gerontologist*. 2011;51(6):822-832.

9. McMahon S, Fleury J. External validity of physical activity interventions for community-dwelling older adults with fall risk: A quantitative systematic literature review. *J Adv Nurs. 2012. 68(10), 2140-2154.*

10. Thomas S, Mackintosh S, Halbert J. Does the 'Otago exercise programme'reduce mortality and falls in older adults?: A systematic review and meta-analysis. *Age Ageing*. 2010;39(6):681-687.

11. El-Khoury F, Cassou B, Charles MA, Dargent-Molina P. The effect of fall prevention exercise programmes on fall induced injuries in community dwelling older adults: Systematic review and meta-analysis of randomised controlled trials. *BMJ*. 2013; 347:f6234.

12. Robertson MC, Devlin N, Gardner MM, Campbell AJ. Effectiveness and economic evaluation of a nurse delivered home exercise programme to prevent falls. 1: Randomised controlled trial. *BMJ: British Medical Journal*. 2001;322(7288):697.

13. Kyrdalen IL, Moen K, Røysland AS, Helbostad JL. The Otago exercise program performed as group training versus home training in fall-prone older people: A randomized controlled trial. *Physiotherapy Research International.* 2013. *19*(2), 108-116.

14. Ainsworth B, Cahalin L, Buman M, Ross R. The current state of physical activity assessment tools. *Prog Cardiovasc Dis*. 2015;57(4):387-395.

15. Baert V, Gorus E, Mets T, Geerts C, Bautmans I. Motivators and barriers for physical activity in the oldest old: A systematic review. *Ageing research reviews*. 2011;10(4):464-474.

16. Greaney ML, Lees FD, Blissmer BJ, Riebe D, Clark PG. Psychosocial factors associated with physical activity in older adults. *Annual Review of Gerontology and Geriatrics*. 2016;36(1):273-291.

17. Mathews AE, Laditka SB, Laditka JN, et al. Older adults' perceived physical activity enablers and barriers: A multicultural perspective*. Journal of Aging & Physical Activity.* 2010; 18(2):119-140.

18. Schutzer KA, Graves BS. Barriers and motivations to exercise in older adults. *Prev Med*. 2004;39(5):1056-1061.

19. McAuley E, Blissmer B, Marquez DX, Jerome GJ, Kramer AF, Katula J. Social relations, physical activity, and well-being in older adults. *Prev Med*. 2000; 31(5):608-617.

20. van Stralen MM, De Vries H, Mudde AN, Bolman C, Lechner L. Determinants of initiation and maintenance of physical activity among older adults: A literature review. *Health Psychology Review*. 2009;3(2):147-207.

21. Rhodes RE, Martin AD, Taunton JE, Rhodes EC, Donnelly M, Elliot J. Factors associated with exercise adherence among older adults. an individual perspective. *Sports Medicine*. 1999;28(6):397-411.

22. Fleury J. The index of readiness: Development and psychometric analysis. *J Nurs Meas*. 1994;2(2):143-154.

23. Harkin B, Webb TL, Chang BP, et al. Does monitoring goal progress promote goal attainment? A meta-analysis of the experimental evidence. *Psychological Bulletin.* 2016; 142, (2): 198

24. Vohs KD, Baumeister RF. Understanding self-regulation: An introduction. *Handbook of self-regulation: Research, theory, and applications*. 2004:1-9.

25. Perez A, Fleury J. Wellness motivation theory in practice. *Geriatric nursing (New York, NY)*. 2009;30(2 Suppl):15.

26. Floegel TA, Giacobbi Jr PR, Dzierzewski JM, et al. Intervention markers of physical activity maintenance in older adults. *Am J Health Behav*. 2015;39(4):487-499.

27. King AC, Friedman R, Marcus B, et al. Ongoing physical activity advice by humans versus computers: The community health advice by telephone (CHAT) trial. *Health Psychology*. 2007;26 (6):718.

28. Babazono A, Kame C, Ishihara R, Yamamoto E, Hillman AL. Patient-motivated prevention of lifestyle-related disease in japan. *Disease Management & Health Outcomes*. 2007;15(2):119-126.

29. Fleig L, McAllister MM, Chen P, et al. Health behaviour change theory meets falls prevention: Feasibility of a habit-based balance and strength exercise intervention for older adults. *Psychol Sport Exerc*. 2016;22:114-122.

30. McTiernan A, Sorensen B, Irwin ML, et al. Exercise effect on weight and body fat in men and women. *Obesity*. 2007;15(6):1496-1512.

31. Chase JA. Interventions to increase physical activity among older adults: A meta-analysis. *Gerontologist.* 2015 Aug; 55(4):706-18.

32. O'Brien N, McDonald S, Araújo-Soares V, et al. The features of interventions associated with long-term effectiveness of physical activity interventions in adults aged 55 to 70 years: A systematic review and meta-analysis. *Health Psychology Review. 2015 9(4), 417-433..*
33. French DP, Olander EK, Chisholm A, Mc Sharry J. Which behaviour change techniques are most effective at increasing older adults' self-efficacy and physical activity behaviour? A systematic review. *Annals of Behavioral Medicine. 2014; 48(2), 225-234.*

34. Collins LM. Design of experiments with multiple independent variables: A resource management perspective on complete and reduced factorial designs. *Psychol Methods*. 2009;14(3):202.

35. Collins LM, Dziak JJ, Kugler KC, Trail JB. Factorial experiments: Efficient tools for evaluation of intervention components. *Am J Prev Med*. 2014;47(4):498-504.

36. McMahon S, Vankipuram M, Hekler E, Fleury J. Design and evaluation of theory-based technology to enhance motivation for physical activity. *Translational behavioral medicine* 2014: *4*(1), 95-107.

37. Patel MS, Asch DA, Volpp KG. Wearable devices as facilitators, not drivers, of health behavior change. *JAMA*. 2015;313(5):459-460.

38. Adam Noah J, Spierer DK, Gu J, Bronner S. Comparison of steps and energy expenditure assessment in adults of fitbit tracker and ultra to the actical and indirect calorimetry. *J Med Eng Technol*. 2013;37(7):456-462.

39. Takacs J, Pollock CL, Guenther JR, Bahar M, Napier C, Hunt MA. Validation of the Fitbit One activity monitor device during treadmill walking. *Journal of Science and Medicine in Sport*. 2014;17(5):496-500.

40. Diaz KM, Krupka DJ, Chang MJ, et al. Fitbit®: An accurate and reliable device for wireless physical activity tracking. *Int J Cardiol*. 2015;185:138-140.

41. Paul SS, Tiedemann A, Hassett LM, et al. Validity of the fitbit activity tracker for measuring steps in community-dwelling older adults. *BMJ Open Sport & Exercise Medicine*. 2015;1(1).

42. Clinical and Translational Science Awards Consortium, Community Engagement Key Function Committee. *Principles of community engagement.* Department of Health & Human Services, National Institutes of Health, Centers for Disease Control and Prevention, Agency for Toxic Substances and Disease Registry, Clinical and Translational Science Awards; 2011.

43. French SD, Green SE, O'Connor DA, et al. Developing theory-informed behaviour change interventions to implement evidence into practice: A systematic approach using the theoretical domains framework. *Implement Sci. 2012; 7:38-5908-7-38.*

44. Collins LM. The multiphase optimization strategy for engineering effective tobacco use interventions. *Annals of behavioral medicine*. 2011;41(2):208.

45. Riddle M, Science of Behavior Change Working Group. News from the NIH: Using an experimental medicine approach to facilitate translational research. *Translational behavioral medicine*. 2015;5(4):486.

46. Carstensen LL. The influence of a sense of time on human development. *Science*. 2006; 312(5782):1913-1915.

47. Carstensen LL, Pasupathi M, Mayr U, Nesselroade JR. Emotional experience in everyday life across the adult life span. *J Pers Soc Psychol*. 2000;79(4):644.

48. Hobbs N, Godfrey A, Lara J, et al. Are behavioral interventions effective in increasing physical activity at 12 to 36 months in adults aged 55 to 70 years? A systematic review and meta-analysis. *BMC Med*. 2013; 11:75-7015-11-75.

49. McMahon SK, Wyman JF, Belyea MJ, Shearer N, Hekler EB, Fleury J. Combining motivational and physical intervention components to promote fall-reducing physical activity among community-dwelling older adults: A feasibility study. *Am J Health Promot ajhp.130522-ARB-265, first published on June 16, 2016 doi:10.4278/ajhp.130522-ARB-265*

50. McMahon S, Lewis B, Oakes JM, Guan W, Wyman J, Rothman J. Older adults' experiences using a wearable device for tracking physical activity. *JMIR Mhealth Uhealth 2016;4(2):e35*.

51. Cain KL, Geremia CM. Accelerometer data collection and scoring manual. . 2011.

52. Biernacki P, Waldorf D. Snowball sampling: Problems and techniques of chain referral sampling. *Sociological methods & research*. 1981;10(2):141-163.

53. Kiernan M, Crane MM, Voils CI, Leahey TM, Spring B. Evidence-based strategies that improve recruitment, adherence, and retention of randomized trials. .*Annals of Behavioral Medicine*. 2015; 49:S42-S42.

54. Topolski TD, LoGerfo J, Patrick DL, Williams B, Walwick J, Patrick MB. The rapid assessment of physical activity (RAPA) among older adults. *Prev Chronic Dis*. 2006;3(4):A118.

55. Stevens JA, Phelan EA. Development of STEADI: A fall prevention resource for health care providers. *Health Promot Pract*. 2013; 14(5):706-714.

56. Resnick B, Ory MG, Hora K, et al. A proposal for a new screening paradigm and tool called exercise assessment and screening for you (EASY). *J Aging Phys Act*. 2008;16(2):215-233.

57. Chodzko-Zajko WJ, Resnick B, Ory MG. Beyond screening: Tailoring physical activity options with the EASY tool. *Translational behavioral medicine*. 2012;2(2):244-248.

58. Newkirk LA, Kim JM, Thompson JM, Tinklenberg JR, Yesavage JA, Taylor JL. Validation of a 26-point telephone version of the mini-mental state examination. *J Geriatr Psychiatry Neurol*. 2004;17(2):81-87.

59. Nelson ME, Rejeski WJ, Blair SN, et al. Physical activity and public health in older adults: Recommendation from the american college of sports medicine and the American heart association. *Med Sci Sports Exerc*. 2007;39(8):1435.

60. Hannan PJ. Experimental social epidemiology—controlled community trials. *Methods in social epidemiology*. Oakes, J. Michael, and Jay S. Kaufman, eds. 2006:341-369.006:341-369.

61. Stevens JA. *A CDC compendium of effective fall interventions: What works for community-dwelling older adults.* Centers for Disease Control and Prevention. National Center for Injury Prevention and Control. Division of Unintentional Injury Prevention; http://www.cdc.gov/homeandrecreationalsafety/falls/compendium.html2015.

62. U.S. Department of Health and Human Services. National Institute on Aging, Health and Aging. https://www.nia.nih.gov/health. Updated 2016.

63. Herman SW. Go4life:< Http://Go4life. NIA. NIH. gov>. *Journal of Consumer Health On the Internet*. 2014;18(3):271-278.

64. Gardner MM, Buchner DM, Robertson MC, Campbell AJ. Practical implementation of an exercise-based falls prevention programme. *Age Ageing*. 2001;30(1):77-83.

65. Thompson WG, Kuhle CL, Koepp GA, McCrady-Spitzer SK, Levine JA. "Go4Life" exercise counseling, accelerometer feedback, and activity levels in older people. *Arch Gerontol Geriatr*. 2014;58(3):314-319.

66. World Health Organization. Global recommendations on physical activity for health. 2010. http://www.who.int/dietphysicalactivity/factsheet_olderadults/en/

67. Fitabase. Research platform. http://www.fitabase.com/ Updated 2016.

68. Bellg AJ, Borrelli B, Resnick B, et al. Enhancing treatment fidelity in health behavior change studies: Best practices and recommendations from the NIH behavior change consortium. *Health Psychology*. 2004;23(5):443.

69. Glasgow RE, Lichtenstein E, Marcus AC. Why don't we see more translation of health promotion research to practice? rethinking the efficacy-to-effectiveness transition. *Am J Public Health*. 2003;93(8):1261-1267.

70. Buman MP, Hekler EB, Haskell WL, et al. Objective light-intensity physical activity associations with rated health in older adults. *American Journal of Epidemiology*. 2010;172(10):1155-1165.

71. Hawley-Hague H, Horne M, Skelton DA, Todd C. Review of how we should define (and measure) adherence in studies examining older adults' participation in exercise classes. *BMJ Open*. 2016;6(6):e011560-2016-011560.

72. Washburn, R. A., Smith, K. W., Jette, A. M., & Janney, C. A. (1993). The Physical Activity Scale for the Elderly (PASE): development and evaluation. Journal of clinical epidemiology, 46(2), 153-162.

73. Lamb SE, Jørstad-Stein EC, Hauer K, Becker C. Development of a common outcome data set for fall injury prevention trials: The prevention of falls network europe consensus. *J Am Geriatr Soc*. 2005;53(9):1618-1622.

74. Hauer K, Lamb SE, Jorstad EC, Todd C, Becker C. Systematic review of definitions and methods of measuring falls in randomised controlled fall prevention trials. *Age Ageing*. 2006;35(1):5-10.

75. Kunkel D, Pickering RM, Ashburn AM. Comparison of retrospective interviews and prospective diaries to facilitate fall reports among people with stroke. *Age Ageing*. 2011;40(2):277-280.

76. Hays RD, Bjorner JB, Revicki DA, Spritzer KL, Cella D. Development of physical and mental health summary scores from the patient-reported outcomes measurement information system (PROMIS) global items. *Quality of Life Research*. 2009; 18(7):873-880.

77. Guralnik JM, Simonsick EM, Ferrucci L, et al. A short physical performance battery assessing lower extremity function: Association with self-reported disability and prediction of mortality and nursing home admission. *J Gerontol*. 1994; 49(2):M85-M94.

78. Cella D, Riley W, Stone A, et al. The patient-reported outcomes measurement information system (PROMIS) developed and tested its first wave of adult self-reported health outcome item banks: 2005–2008. *J Clin Epidemiol*. 2010; 63(11):1179-1194.

79. Rose M, Bjorner JB, Becker J, Fries J, Ware J. Evaluation of a preliminary physical function item bank supported the expected advantages of the patient-reported outcomes measurement information system (PROMIS). *J Clin Epidemiol*. 2008; 61(1):17-33.

80. Sallis JF, Grossman RM, Pinski RB, Patterson TL, Nader PR. The development of scales to measure social support for diet and exercise behaviors. *Prev Med*. 1987; 16(6):825-836.

81. Resnick B, Jenkins LS. Testing the reliability and validity of the self-efficacy for exercise scale. *Nurs Res*. 2000; 49(3):154-159.

82. Wilson PM, Sabiston CM, Mack DE, Blanchard CM. On the nature and function of scoring protocols used in exercise motivation research: An empirical study of the behavioral regulation in exercise questionnaire. *Psychol Sport Exerc*. 2012; 13(5):614-622.

83. Markland D, Tobin V. A modification to the behavioural regulation in exercise questionnaire to include an assessment of amotivation. *Journal of Sport and Exercise Psychology*. 2004; 26(2):191-196.

84. Fleury J. The index of self-regulation: Development and psychometric analysis. *J Nurs Meas*. 1998;6(1):3-17.

85. Kiresuk TJ, Smith A, Cardillo JE. *Goal attainment scaling: Applications, theory, and measurement.* Psychology Press; 2014.

86. Rockwood K, Howlett S, Stadnyk K, Carver D, Powell C, Stolee P. Responsiveness of goal attainment scaling in a randomized controlled trial of comprehensive geriatric assessment. *J Clin Epidemiol*. 2003; 56(8):736-743.

87. Leveille SG, Jones RN, Kiely DK, et al. Chronic musculoskeletal pain and the occurrence of falls in an older population. *JAMA*. 2009; 302(20):2214-2221.

88. Rubenstein LZ. Falls in older people: Epidemiology, risk factors and strategies for prevention. *Age Ageing*. 2006; 35(suppl 2):ii37-ii41.

89. Mendoza T, Mayne T, Rublee D, Cleeland C. Reliability and validity of a modified brief pain inventory short form in patients with osteoarthritis. *European Journal of Pain*. 2006; 10(4):353-353.

90. Tan G, Jensen MP, Thornby JI, Shanti BF. Validation of the brief pain inventory for chronic nonmalignant pain. *The Journal of Pain*. 2004;5(2):133-137.

91. American Geriatrics Society 2012 Beers Criteria Update Expert Panel. American geriatrics society updated beers criteria for potentially inappropriate medication use in older adults. *J Am Geriatr Soc*. 2012; 60(4):616-631.

92. Conn VS, Valentine JC, Cooper HM. Interventions to increase physical activity among aging adults: A meta-analysis. *Annals of Behavioral Medicine*. 2002; 24(3):190-200.

93. Tudor-Locke C, Craig CL, Aoyagi Y, et al. How many steps/day are enough for older adults and special populations. *Int J Behav Nutr Phys Act*. 2011;8(1):80.

94. Dziak, J. J., Collins, L. M., & Wagner, A. T. FactorialPowerPlan SAS macro suite users' guide (version 1.0). University Park: The Methodology Center, Penn State. . http://methodology.psu.edu.

95. Harris PA, Taylor R, Thielke R, Payne J, Gonzalez N, Conde JG. Research electronic data capture (REDCap)—a metadata-driven methodology and workflow process for providing translational research informatics support. *J Biomed Inform*. 2009; 42(2):377-381.

96. MacKinnon DP, Luecken LJ. How and for whom? Mediation and moderation in health psychology. *Health Psychol*. 2008; 27(2 Suppl):S99-S100.

97. Valeri L, VanderWeele TJ. Mediation analysis allowing for exposure–mediator interactions and causal interpretation: Theoretical assumptions and implementation with SAS and SPSS macros. *Psychol Methods*. 2013; 18(2):137.

98. Rothwell PM. Subgroup analysis in randomised controlled trials: Importance, indications, and interpretation. *The Lancet*. 2005;365(9454):176-186.

99. Burke JF, Sussman JB, Kent DM, Hayward RA. Three simple rules to ensure reasonably credible subgroup analyses. *BMJ*. 2015;351:h5651.

100. Bell ML, Kenward MG, Fairclough DL, Horton NJ. Differential dropout and bias in randomised controlled trials: When it matters and when it may not. *BMJ*. 2013;346:e8668.

101. Metropolitan Area Agency on Aging. **Falls prevention day event on September 23**. http://metroaging.org/falls-prevention-september-23/.

102. Center for Disease Control and Prevention. National Center for Health Statistics. *Health Indicators Warehouse*. http://www.healthindicators.gov/

103. Freid VM, Bernstein AB, Bush MA. Multiple chronic conditions among adults aged 45 and over: Trends over the past 10 years. *Women*. 2012; 45:64.

104. Abdulla A, Adams N, Bone M, et al. Guidance on the management of pain in older people. *Age Ageing*. 2013;42 Suppl 1:i1-57.

105. Qato DM, Wilder J, Schumm LP, Gillet V, Alexander GC. Changes in prescription and over-the-counter medication and dietary supplement use among older adults in the United States, 2005 vs 2011. *JAMA internal medicine*. 2016;176(4):473-482.

106. U.S. Census Bureau. State and county quick facts. http://www.census.gov/quickfacts/table/PST045215/00.

107. Hebert LE, Weuve J, Scherr PA, Evans DA. Alzheimer disease in the United States (2010-2050) estimated using the 2010 census. *Neurology*. 2013; 80(19):1778-1783.

108. US Department of Health and Human Services. *Administration on Aging. A profile of older Americans: 2014*. 2014.

109. McMahon, S. K., Lewis, B., Oakes, J. M., Wyman, J. F., Guan, W., & Rothman, A. J. (2017). Assessing the effects of interpersonal and intrapersonal behavior change strategies on physical activity in older adults: A factorial experiment. *Annals of Behavioral Medicine*, *51*(3), 376-390.

110. Cawthon, P. M., Orwoll, E. S., Ensrud, K. E., Cauley, J. A., Kritchevsky, S. B., Cummings, S. R., & Newman, A. (2020). Assessing the impact of the covid-19 pandemic and accompanying mitigation efforts on older adults. *The Journals of Gerontology: Series A*, *75*(9), e123-e125.

111. Baker, P. S., Bodner, E. V., & Allman, R. M. (2003). Measuring life-space mobility in community-dwelling older adults. Journal of the American Geriatrics Society, 51(11), 1610-1614.

112. Glasgow, R. E., Toobert, D. J., Barrera Jr, M., & Strycker, L. A. (2005). The Chronic Illness Resources Survey: cross-validation and sensitivity to intervention. *Health Education Research*, *20*(4), 402-409.

113. Callahan, C. M., Unverzagt, F. W., Hui, S. L., Perkins, A. J., & Hendrie, H. C. (2002). Six-item screener to identify cognitive impairment among potential subjects for clinical research. Medical care, 771-781.
