# Supplementary material for: Effect of Intrapersonal and Interpersonal Behavior Change Strategies on Physical Activity Among Older Adults: A Randomized Clinical Trial
Source: JAMA Netw Open. 2024 Feb 29;7(2):e240298. doi: 10.1001/jamanetworkopen.2024.0298 (PMC10905305; doi:10.1001/jamanetworkopen.2024.0298)
Supplement: Supplement 2. — eTable 1. Baseline Value Adjusted 2 (Intrapersonal: Yes, No) x 2 (Interpersonal: Yes, No) ANCOVA Results for Change in Physical Activity Outcomes Postintervention at 1 Week, 6 Months, and 12 Months eTable 2. Comparison of Baseline Characteristics and Physical Activity Outcome Values Among All Participants (Overall), Those Who Were Randomized With a Partner, and Those Who Were Not Randomized With a Partner eFigure. Longitudinal Analysis of Effects of Interpersonal Behavior Change Strategies on Primary Outcome of Average Daily Minutes of Total Physical Activity eTable 3. Potential Clustering Effects eReferences [file jamanetwopen-e240298-s002.pdf]

Supplemental Online Content

McMahon SK, Lewis BA, Guan W, et al. Effect of intrapersonal and interpersonal behavior change strategies on physical activity among older adults: a randomized clinical trial. *JAMA Netw Open*. 2024;7(2):e240298. doi:10.1001/jamanetworkopen.2024.0298

eTable 1. Baseline Value Adjusted 2 (Intrapersonal: Yes, No) x 2 (Interpersonal: Yes, No) ANCOVA Results for Change in Physical Activity Outcomes Postintervention at 1 Week, 6 Months, and 12 Months
eTable 2. Comparison of Baseline Characteristics and Physical Activity Outcome Values Among All Participants (Overall), Those Who Were Randomized With a Partner, and Those Who Were Not Randomized With a Partner
eFigure 1. Longitudinal Analysis of Effects of Interpersonal Behavior Change Strategies on Primary Outcome of Average Daily Minutes of Total Physical Activity
eTable 3. Potential Clustering Effects
eReferences

This supplemental material has been provided by the authors to give readers additional information about their work.

© 2024 McMahon SK et al. *JAMA Network Open*.

eTable 1. Baseline Value Adjusted 2 (Intrapersonal: Yes, No) x 2 (Interpersonal: Yes, No) ANCOVA Results for Change in Physical Activity Outcomes Postintervention at 1 Week, 6 Months, and 12 Months

**PA Outcomes**	**Difference in PA outcome adjusted for its baseline value**					
	**1 week post-intervention**		**6 months post-intervention**		**12 months post-intervention**	
	Estimate (95% CI)	P	Estimate (95% CI)	P	Estimate (95% CI)	P
**Average daily minutes of total PA[a]**						
Baseline value	0.84 (0.77, 0.91)	<.0001	0.83 (0.75, 0.91)	<.0001	0.89 (0.81, 0.97)	<.0001
Intrapersonal BCS: Yes	1.82 (8.55, 12.18)	.73	-3.93 (-14.99, 7.14)	.49	-8.77 (-20.47, 2.94	.14
Interpersonal BCS: Yes	27.05 (17.15, 36.96)	<.0001	20.77 (9.96, 31.58)	.0002	27.51 (16.19, 38.83)	<.0001
Interaction	12.48 (-7.32, 32.27)	0.22	11.96 (-9.65, 33.58)	.277	7.25 (-15.33, 29.82)	.53
**Average daily step count[a]**						
Baseline value	0.97 (0.90, 1.03)	<.0001	1.01 (0.94, 1.08)	<.0001	0.96 (0.88, 1.05)	<.0001
Intrapersonal BCS: Yes	44.00 (-244.5, 332.5)	.76	-65.40 (-368.7, 237.9)	.67	-240.7 (-614.5, 133.1)	.21
Interpersonal BCS: Yes	1058 (796.2, 1320.7)	<.0001	775.9 (485.8, 1065.9)	<.0001	925.7 (566.3, 1285.1)	<.0001
Interaction	352.1 (-172.7, 876.99)	.19	275.68 (-305.6 856.99)	.35	209.9 (-508.9, 928.8)	.57
**Average daily minutes of MVPA[a]**						
Baseline value	0.63 (0.53, 0.73)	<.0001	0.52 (0.42, 0.61)	<.0001	0.79 (0.69, 0.90)	<.0001
Intrapersonal BCS: Yes	0.33 (-5.37, 6.04)	.91	-2.05 (-7.59, 3.48)	.47	-6.05 (-12.26, 0.16)	.06
Interpersonal BCS: Yes	9.31 (3.71, 14.92)	.0012	7.60 (2.13, 13.07)	.007	11.64 (5.54, 17.74)	.0002
Interaction	5.72 (-9.48, 13.02)	.76	5.56 (-5.21, 16.69)	.30	-2.85 (-15.02, 9.32)	.64
**Self-reported PA (PASE) [b]**						
Baseline value	0.71 (0.61, 0.81)	<.0001	0.67 (0.56, 0.78)	<.0001	0.75 (0.62, 0.88)	<.0001
Intrapersonal BCS: Yes	5.00 (-5.18, 15.18)	.33	2.24 (-9.15,13.63)	.70	6.19 (-7.24, 19.61)	.37
Interpersonal BCS: Yes	5.87 (-4.30, 16.05)	.26	3.26 (-8.12, 14.64)	.57	0.04 (-13.40,13.48)	.99
Interaction	2.23 (-18.20, 22.66)	.83	-10.38 (-33.24, 12.47)	.37	-6.14 (-33.13, 20.85)	.65

Notes: PA = physical activity; BCS = behavior change strategy; Interaction = interaction effects of receiving intrapersonal and interpersonal components, adjusting for the other, on each PA metric and time point

© 2024 McMahon SK et al. *JAMA Network Open*.

eTable 2. Comparison of Baseline Characteristics and Physical Activity Outcome Values Among All Participants (Overall), Those Who Were Randomized With a Partner, and Those Who Were Not Randomized With a Partner

	Overall (N=309)	Participants randomized with a partner (N=76)	Participants not randomized with a partner (N=233)
**Age, mean (SD), y**	77.4 (5.0)	77.9 (5.1)	77.2 (5.0)
**Sex, N (%)**			
Male	69 (22.3%)	24 (31.6%)	45 (19.3%)
Female	240 (77.7%)	52 (68.4%)	188 (80.7%)
**Race, N (%)**			
Black/ African American	48 (15.5%)	12 (15.8%)	36 (15.5%)
Other	6 (1.9%)	1 (1.3%)	5 (2.1%)
White	255 (82.5%)	63 (82.9%)	192 (82.4%)
**Hispanic, N (%)**	7 (2.3%)	3 (3.9%)	4 (1.7%)
**Education, N (%)**			
HS graduate or less	38 (12.3%)	10 (13.2%)	28 (12.0%)
Some college / technical school	86 (27.8%)	20 (26.3%)	66 (28.3%)
College graduate or higher	185 (59.9%)	46 (60.5%)	139 (59.7%)
**Living Situation, mean (SD)**			
N living in household, mean (SD)	1.7 (0.8)	1.8 (0.7)	1.6 (0.8)
Participated during COVID-19, N[1] (%)	179 (57.9%)	38 (50.0%)	141 (60.5%)
**Health, mean (SD)**			
Pain intensity[2]	1.6 (1.9)	1.7 (1.9)	1.6 (2.0)
Interference from pain[2]	1.0 (1.7)	0.9 (1.6)	1.0 (1.7)
Average # chronic conditions[3]	2.1 (1.2)	2.3 (1.2)	2.1 (1.1)
Global physical health[4]	39.4 (5.1)	39.2 (5.2)	39.5 (5.0)
Global mental health[4]	52.0 (7.8)	51.6 (8.4)	52.1 (7.7)
**Physical Activity, mean (SD)**			
Daily minutes of total PA[5]	177 (71)	177 (63)	177 (74)
Daily step count[5]	4227 (2267)	4466 (2367)	4148 (2233)
Daily minutes of MVPA[5]	21 (29)	22 (22)	21 (31)
PASE[6]	105.7 (52.1)	99.1 (51.8)	107.8 (52.1)

Notes: PA=Physical activity, MPVA=Moderate + Vigorous Physical Activity

1=Participated in Ready Steady3.0 during the initial phase of the pandemic, between March 2020 and May 2021

2= Brief Pain Inventory—Short Form, a 9 item self-report questionnaire used to evaluate the average intensity and impact of a person's pain, scores ranging from 0 to 10.[1]

3 = Chronic conditions via self-report

4 = Patient-Reported Outcomes Measurement Information System (PROMIS) Scale v1.1--Global Health, standardized scores based on US adult population, including older adults, with average scores of 50, and scores > 50 representing greater self-ratings of physical or mental health.[2]

5= PA metrics measured via accelerometers in participants' wearable activity monitors (Fitbit Charge 2), averaged over 7 to 10 days. Validation criteria included participants wearing the monitor for a minimum of 4 days, at least 10 hours per day.

6 = Physical Activity Scale for Elderly, scores ranging from 0 to >400, based on self-report.[3]

© 2024 McMahon SK et al. *JAMA Network Open*.

eFigure 1. Longitudinal Analysis of Effects of Interpersonal Behavior Change Strategies on Primary Outcome of Average Daily Minutes of Total Physical Activity

Per protocol, we conducted linear mixed models to examine the variation of interpersonal component effects on average daily minutes of total physical activity across assessment time points, to confirm that the effects did not change significantly post-treatment This model included a participant-level random intercept to account for repeated observations, terms for differences between the two groups (assignment to interpersonal, no vs yes), and group-time interactions with adjustment for baseline average daily minutes of total physical activity.

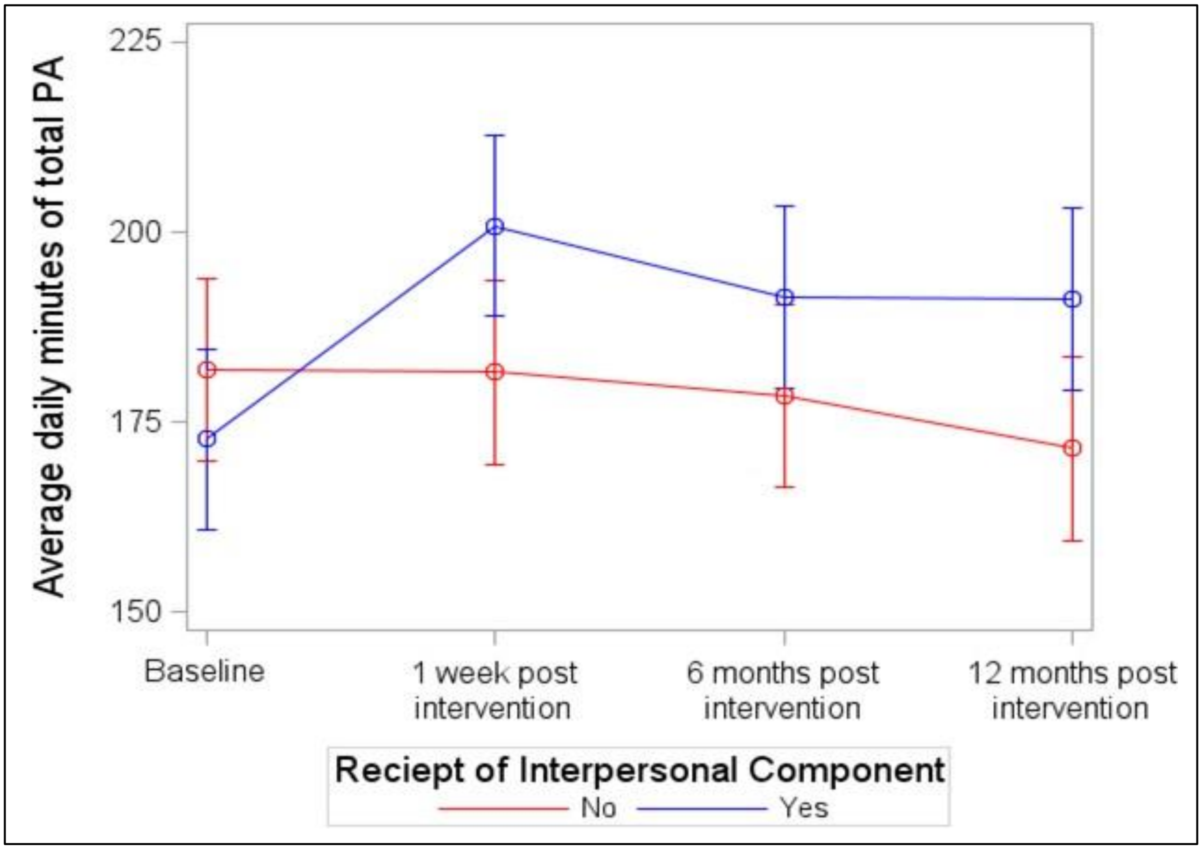

Notes: Results from linear mixed models showed significant effects of the interpersonal component over time on average (least square means and 95% CI) of daily minutes of total physical activity. Model results confirmed (F [3, 904] =14.3, P= <.0001) that participants who received this component had significantly greater mean physical activity minutes at all post-intervention follow up times, compared to those who did not receive to this component.

© 2024 McMahon SK et al. *JAMA Network Open*.

eTable 3. Potential Clustering Effects

The minority of participants in Ready Steady 3.0 were randomized together with a partner, while all received the intervention with 4 to 5 other participants who were randomized to their small group. We assumed the observed outcomes within pairs and small groups would not be correlated, based on prior research.[4,5] To confirm this assumption, we estimated potential clustering effects of the Interpersonal Behavior Change Strategies on the primary outcome of daily minutes of total physical activity, averaged over 7 to 10 days at each time point. To do this we used linear mixed models as extensions of the single level models used in the main ANCOVA analyses, that were adjusted for baseline values of PA, and included random intercepts representing whether participants were randomized with a partner (yes, no), nested within intervention small group (1 to 52) to which participants were assigned.

	**Difference in average daily minutes of total physical activity, adjusted for its baseline value and accounting for potential clustering effects of being randomized with a partner, and small group to which a participant was randomized**					
	**1-week post-intervention**		**6 months post-intervention**		**12 months post-intervention**	
	**Estimate (SE)**	**P**	**Estimate (SE)**	**P**	**Estimate (SE)**	**P**
**Baseline PA**	0.85 (.04)	<.0001	0.84 (.04)	<.0001	0.89 (04)	<.0001
**Partner (intercept)**	294.64 (426.89)	.25	312.96 (429.32)	.23	122.62 (110.51)	.13
**Small Group No.**	105.39 (87.79)	.12	156.68 (111.13)	.08	120.36 (112.56)	.14
**Interpersonal BCS: Yes**	26.73 (5.76)	<.0001	20.58 (6.43)	.003	27.76 (6.32)	<.0001

© 2024 McMahon SK et al. *JAMA Network Open*.

eReferences

1. Mendoza T, Mayne T, Rublee D, Cleeland C. Reliability and validity of a modified Brief Pain Inventory short form in patients with osteoarthritis. *European Journal of Pain*. 2006;10(4):353.

2. Hays, RD, Bjorner, J, Revicki, R A, Spritzer, K L, & Cella, D. Development of physical and mental health summary scores from the Patient Reported Outcomes Measurement Information System (PROMIS) global items. *Quality of Life Research*. 2009; 18(7),873-80. (PMCID: PMC2724630)

3. Washburn RA, McAuley E, Katula J, Mihalko SL, Boileau RA. The Physical Activity Scale for the Elderly (PASE): evidence for validity. *J Clin Epidemiol*. 1999; 52(7):643-651. Medline:10391658 7 doi:10.1016/S0895-4356(99)00049-9.

4. McMahon SK, Lewis B, Oakes JM, Wyman JF, Guan W, Rothman AJ. Assessing the effects of interpersonal and intrapersonal behavior change strategies on physical activity in older adults: A factorial experiment. *Annals of Behavioral Medicine*. 2017;51(3):376-390. doi:10.1007/s12160-016-9863-z

5. Stathi A, Greaves CJ, Thompson JL, et al. Effect of a physical activity and behaviour maintenance programme on functional mobility decline in older adults: the REACT (Retirement in Action) randomised controlled trial. *The Lancet Public Health*. 2022;7(4):e316-e326. doi:10.1016/S2468-2667(22)00004-4

© 2024 McMahon SK et al. *JAMA Network Open*.
