# Supplementary material for: Effect of Intrapersonal and Interpersonal Behavior Change Strategies on Physical Activity Among Older Adults: A Randomized Clinical Trial
Source: JAMA Netw Open. 2024 Feb 29;7(2):e240298. doi: 10.1001/jamanetworkopen.2024.0298 (PMC10905305; doi:10.1001/jamanetworkopen.2024.0298)
Supplement: Supplement 3. — Data Sharing Statement [file jamanetwopen-e240298-s003.pdf]

Data Sharing Statement

McMahon. Effect of Intrapersonal and Interpersonal Behavior Change Strategies on Physical Activity Among Older Adults. *JAMA Netw Open.* Published February 29, 2024. doi:10.1001/jamanetworkopen.2024.0298

Data
Data available: Yes
Data types: Deidentified participant data, Data dictionary
How to access data: skmcmaho@umn.edu URL of UMN Data Repository (DRUM) will be made available upon publication
When available: With publication

Supporting Documents
Document types: None

Additional Information
Who can access the data: Once in DRUM, data are available for immediate download. Users may contact the author with questions regarding the data.
Types of analyses: Individual level data
Mechanisms of data availability: Users can access data on the UMN DRUM website after publication
Any additional restrictions: Users are expected to abide by the University of Minnesota Acceptable Use Policy, and other University policies, where applicable. However, by using or downloading the data, you signify your agreement to the conditions of use stated below: The user will not make any use of data to identify or otherwise infringe the privacy or confidentiality rights of individuals discovered inadvertently or intentionally in the data. The user will give appropriate attribution to the author(s) of the data in any publication that employs resources provided by the Data Repository. If your use or publication requires permission, you must contact the authors directly; administrators of the Data Repository cannot respond to requests for permission.
